# Opposing cell-type preferences for binding and replication shape influenza A virus infection in human airways

**DOI:** 10.1126/sciadv.aei5782

**Published:** 2026-10-23

**Authors:** Josephine von Kempis, Marie O. Pohl, Elisabeth Gaggioli, Cyrille Niklaus, Alexandra Trkola, Geert-Jan Boons, Robert P. de Vries, Silke Stertz

**Affiliations:** ^1^ Institute of Medical Virology, University of Zurich, Zurich, Switzerland.; ^2^ Department of Chemical Biology and Drug Discovery, Utrecht Institute for Pharmaceutical Sciences, Utrecht University, Utrecht, Netherlands.; ^3^ Complex Carbohydrate Research Center, University of Georgia, Athens, GA, USA.

## Abstract

Influenza A viruses (IAVs) pose a threat to human health. Although receptor specificity, polymerase adaptation, and airway tropism have been studied extensively, it remains unresolved how they combine to shape tropism in airway epithelia. Here, we show that avian IAVs infect ciliated and secretory cells but preferentially bind to ciliated cells because of higher apical α2,3-linked sialic acid levels. As replication levels are comparable between cell types, this translates into overall preference for ciliated cells. Human IAVs also preferentially bind to ciliated cells despite comparable α2,6-linked sialic acid abundance. Instead, they show higher viral RNA and protein levels in secretory cells, linked to their compatibility with human ANP32 proteins and modestly higher ANP32A/B expression in this cell type, resulting in equal infection levels across cell types. Our findings redefine IAV tropism in the human airway as a stage-resolved process shaped by the interplay between apical glycans, viral binding, polymerase cofactor compatibility, and cell-type–specific replication capacity, moving beyond receptor-centric models.

## INTRODUCTION

Influenza A viruses (IAVs) cause respiratory tract infections in humans and represent a major challenge to global health and the economy. Beyond the yearly “flu” season responsible for an estimated 1 billion infections (*1*), causing 3 to 5 million cases of severe illness, and 290,000 to 650,000 deaths annually (*2*), IAVs can pose a pandemic threat (*3*, *4*). This arises from the ability of human IAV to undergo genetic reassortment with viruses from their natural avian reservoir, enabling zoonotic spillover and rapid adaptation to new hosts. Understanding the determinants that govern IAV adaptation to human cells is, therefore, critical for pandemic preparedness.

As the virus enters the human body through the respiratory tract, the airway epithelium is the initial site of viral attachment and infection (*5*). The airway epithelium is a heterogeneous and highly structured microenvironment, composed of a variety of cell subtypes distributed in a polarized fashion along the basal-apical axis: Basal cells differentiate through intermediate stages to secretory cells, responsible for apical mucus production, and subsequently to ciliated cells, which drive mucosal clearance through ciliary beating (*6*–*9*). Additionally, the epithelium also comprises minor cell-type populations, such as ionocytes and tuft cells.

For zoonotic infections to occur in the human airway, avian IAV (aIAV) must overcome multiple species-specific barriers. A variety of these barriers have been characterized (*10*), including differential receptor specificity (*11*), polymerase incompatibility with human ANP32 proteins (*12*), lower pH stability of the envelope protein (*13*, *14*), and sensitivity to antiviral factors such as Mx (*15*, *16*) and BTN3A3 (*17*). At the same time, the initial interaction of the virus with the heterogeneous airway epithelium raises a fundamental question: Which cell subtypes are preferentially infected by different IAV strains, and which host and viral factors affect this process? In 2004, Matrosovich *et al.* (*18*) reported that human-adapted IAV (hIAV) infects predominantly nonciliated cells, whereas aIAV infects mainly ciliated cells. These patterns directly correlated with the distribution of the viral attachment receptors (*19*–*21*): While the avian preferred receptors (α2,3-linked sialic acids) were predominantly detected on ciliated cells, sialic acids with an α2,6-linkage, which are the binding receptors of hIAV, were mainly on nonciliated cells. However, subsequent work has revealed that the infection patterns in the airway epithelium are more complex and less clear-cut than initially suggested. Specifically, both α2,3- and α2, 6-linkages have been detected on ciliated and nonciliated cells (*22*–*24*), and hIAV can also infect or at least bind to ciliated cells (*23*, *25*). More recent work focused on pandemic 2009 H1N1 strains reported that its primary targets are secretory cells, whereas ciliated cells are the secondary targets (*26*), and ultimately that the ciliated cells are the main driver of hIAV H1N1 viral particle production (*24*). Together, these findings reopen the fundamental question regarding the IAV cellular tropism and the underlying viral and cellular determinants.

Here, we reveal that human H1N1 and H3N2 IAVs infect ciliated and secretory cells at comparable levels in the initial infection round in the human bronchial epithelium. In contrast, avian influenza A virus strains (H1N1, H3N8, and H11N6) display a clear preference, but not restriction, for ciliated cells. We further show that the avian virus preferentially binds to ciliated cells, driven by higher abundance of α2,3-linked sialic acids, specifically sialyl Lewis X (sLe^x^) structures, on the apical surface of ciliated cells. We find that human strains also preferentially bind ciliated cells but exhibit higher RNA production in secretory cells due to differential *ANP32* expression levels between the cells, resulting in a dual-tropism phenotype. Last, we show that the observed deficiency in avian virus replication is not driven by the initial up-regulation of interferon-stimulated gene (ISG) transcription but instead by the limited compatibility of the viral polymerase with human ANP32 proteins.

## RESULTS

### IAVs display differential tropism in bronchial epithelial cells

To model the heterogeneity and complexity of the airway epithelium in vitro, we used differentiated primary human bronchial epithelial cells (BEpCs) grown at air-liquid interface (ALI) from three independent donors (donors 1 to 3). The presence of the main bronchial cell types (ciliated, secretory, and basal cells) was determined and quantified using a previously described flow cytometry panel (Fig. 1A) (*27*). We successfully identified all three cell-type populations within each donor (Fig. 1B). To assess the efficiency of the initial infection in BEpCs with a human (hIAV) and an avian influenza A virus (aIAV), we used a seasonal human H1N1 IAV from 2019 (A/Hawaii/70/2019) and an avian H1N1 isolated from a duck (A/Duck/Alberta/35/1976). We infected the cultures with three different multiplicities of infection (MOIs: 1, 5, and 10) as calculated by titration on Madin-Darby canine kidney (MDCK) cells and quantified the percentage of infected cells (NP-positive cells) via immunofluorescent microscopy (Fig. 1C and fig. S1A). At an MOI of 1, ∼10 to 15% of cells were NP positive for both viruses. The proportion of infected cells increased with higher MOI and reached a plateau at ∼30% at an MOI of 5, with no substantial further increase at an MOI of 10. For flow cytometric detection, infected cells were identified by surface hemagglutinin (HA) staining, which showed insufficient sensitivity at 6 hours postinfection (hpi) but a more robust signal at 8 hpi (fig. S1B). To stay within the first replication round while having robust detection of infected cells (fig. S1C), we proceeded with flow cytometric readouts at an MOI of 5 and at 8 hpi for both viral infections across all three donors. For subsequent analysis, we pooled the findings from donors 1 to 3. Contrary to previous results (*18*), both the avian and the human IAVs, infected ciliated and secretory cells (Fig. 1D), whereas infection of basal cells remained minimal. To compare the likelihood of secretory versus ciliated cells becoming infected by a virus while minimizing the effect of between-repeat variability in overall infection levels, we normalized subtype-specific infection rates to the overall percentage of infected cells. We thus yielded a normalized infection rate, which revealed that the avian H1N1 strain has a significant preference to infect ciliated over secretory cells, while the human strain did not display a clear bias toward either subtype (Fig. 1E). We further found that dual tropism for both cell types was true for not only additional H1N1 but also H3N2 human strains, and tropism for ciliated cells could be confirmed for additional avian strains (Fig. 1, E and F). Last, we quantified viral protein levels within the infected cell and found that cells infected with A/Hawaii/70/2019 showed significantly higher viral NP signals than A/Duck/Alberta/35/1976-infected cells (Fig. 1G and fig. S1D). These data indicate that differences in viral protein expression efficiency are evident already at early time points postinfection.

**Fig. 1.**
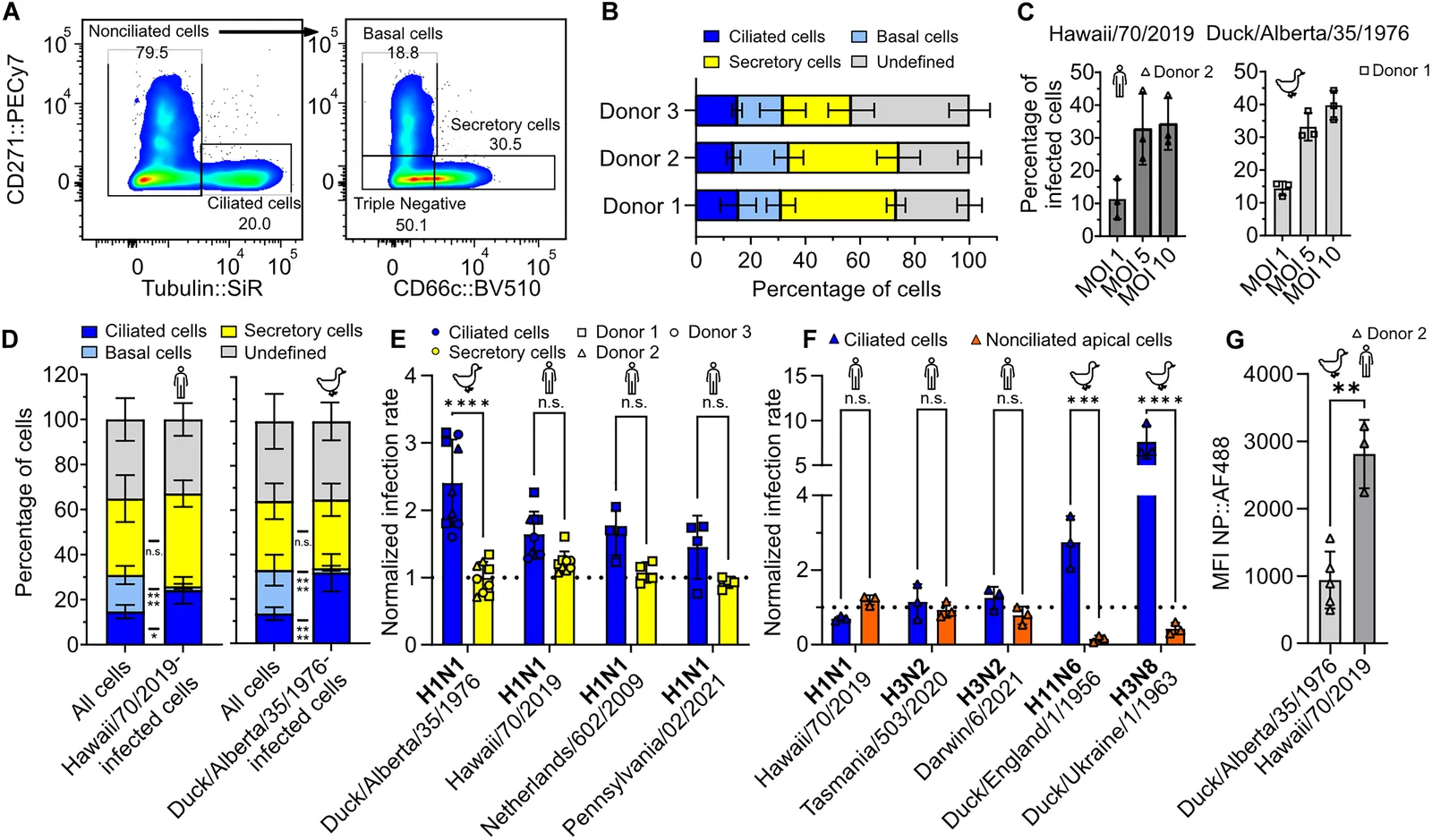
Flow cytometry and microscopy reveal divergent bronchial epithelial cell tropism of human and avian IAVs. (**A**) Representative flow cytometry gating strategy. Cells were stained for microtubules (ciliated cells), CD271 (basal cells), and CD66c (secretory cells). (**B**) Relative cell-type compositions of BEpCs (donors 1 to 3) were determined. Unstained or double-positive cells categorized as “undefined.” (**C**) BEpCs (donors 1 and 2) were infected with A/Duck/Alberta/35/1976 or A/Hawaii/70/2019 with indicated multiplicity of infection (MOI). At 8 hpi, cells were stained for viral NP, tight junctions, nuclei, and infections quantified by microscopy. (**D**) BEpCs were infected with A/Duck/Alberta/35/1976 or A/Hawaii/70/2019 (MOI of 5; 8 hpi) and prepared for flow cytometry. Relative cell-type compositions are shown as percentages of each cell type in total populations and within infected populations. Per donor, values were normalized to the average across experimental repeats. Data from donors 1 to 3 were pooled. One repeat was previously published (*45*). (**E**) Normalized infection rates from (D) and from BEpC donor 1 infected identically with Netherlands/602/2009 or Pennsylvania/02/2021. Data from donors 1 to 3 pooled: donor 1 (square), donor 2 (triangle), and donor 3 (circle). (**F**) BEpCs from donor 2 were infected (MOI of 3) with indicated IAVs. At 7 hpi, cells were stained for NP, ciliated cells, tight junctions, and nuclei. Infections were quantified as percentage of NP-positive cells relative to apical cells and normalized to total infected cells. (**G**) BEpCs (donor 2) infected with Hawaii/70/2019 or Duck/Alberta/35/1976 (MOI of 5) were stained for NP at 7 hpi. Median fluorescence intensity (MFI) of NP in infected cells was quantified. [(B), (C), (F), and (G)] Data represent means ± SD from *n* ≥ 3 independent experiments. [(D) and (E)] Data represent means ± SD from *n* ≥ 3 independent experiments per donor. [(D), (E), and (F)] Statistical significance was determined using two-way analysis of variance (ANOVA) with Šídák’s multiple comparisons test. (G) Statistical significance was determined using unpaired *t* test. *P ≤ 0.05, **P ≤ 0.01, ***P ≤ 0.001, and ****P ≤ 0.0001. n.s., not significant.

To further characterize the cellular tropism during the initial round of viral infection at a transcriptomic level, we performed single-cell RNA sequencing (scRNA-seq). Bronchial epithelial cells from donors 1 to 3 were infected for a total of 6 hours with A/Hawaii/70/2019, A/Duck/Alberta/35/1976, or a mock control. To determine the cell subtypes present in the cultures based on their transcriptomes, we used known canonical cell cluster markers defined as a transcript with at least a 0.25-fold difference (log scale) and an adjusted *P* value of <0.05 between the cells in the tested cluster and the rest of the cells. We distinguished the following epithelial cell subtypes in the cultures: ciliated, secretory, intermediate, suprabasal, basal cells, and ionocytes (Fig. 2A and fig. S2, A and B). All cell subtypes, except suprabasal cells, which were only detected in the bronchial cultures from donor 1, were detected across all three donors (Fig. 2B). The cells were further defined as infected when they expressed transcripts from at least four of the eight viral genome segments, based on previous findings showing that more than 95% of genuinely influenza A virus-infected cells meet this threshold (Fig. 2C) (*28*). With this definition, we aimed to reduce the impact of extracellular viral RNA contamination released by dying or dead cells, without excluding genuinely infected cells. The vast majority of infected cells according to this definition had all eight viral segments present (fig. S3, A to C). For the following sections, we will refer to “infected cells,” when we consider cells with at least four viral segments, while we will refer to the “infected sample” when speaking of all cells of the sample independent of their infectious status. Using this criterion, both viruses displayed consistent infection levels across donors, with around 8% infected cells for the avian strain and 10 to 14% for the human strain (Fig. 2D). The infection composition revealed that the human strain infected roughly equal proportions of ciliated and secretory cells (Fig. 2E), whereas the avian strain infected a larger fraction of ciliated and a smaller fraction of secretory cells. Consistent with our previous findings, only a small percentage of the basal and suprabasal cell populations were infected by both viruses. The same infection patterns were also evident when analyzing each viral segment individually (figs. S3, D and E, and S4, A and B). The normalized infection rates revealed a highly significant preference for ciliated cells for the avian virus and a statistically nonsignificant trend for ciliated cells for the human virus (Fig. 2F). These results are consistent with the flow cytometry and microscopy data (Fig. 1, E and F). Additionally, we found that the overall copy number of viral mRNA in the hIAV-infected samples was 10-fold higher than in the aIAV-infected samples (Fig. 2G), highlighting differences in viral RNA production efficiency.

**Fig. 2.**
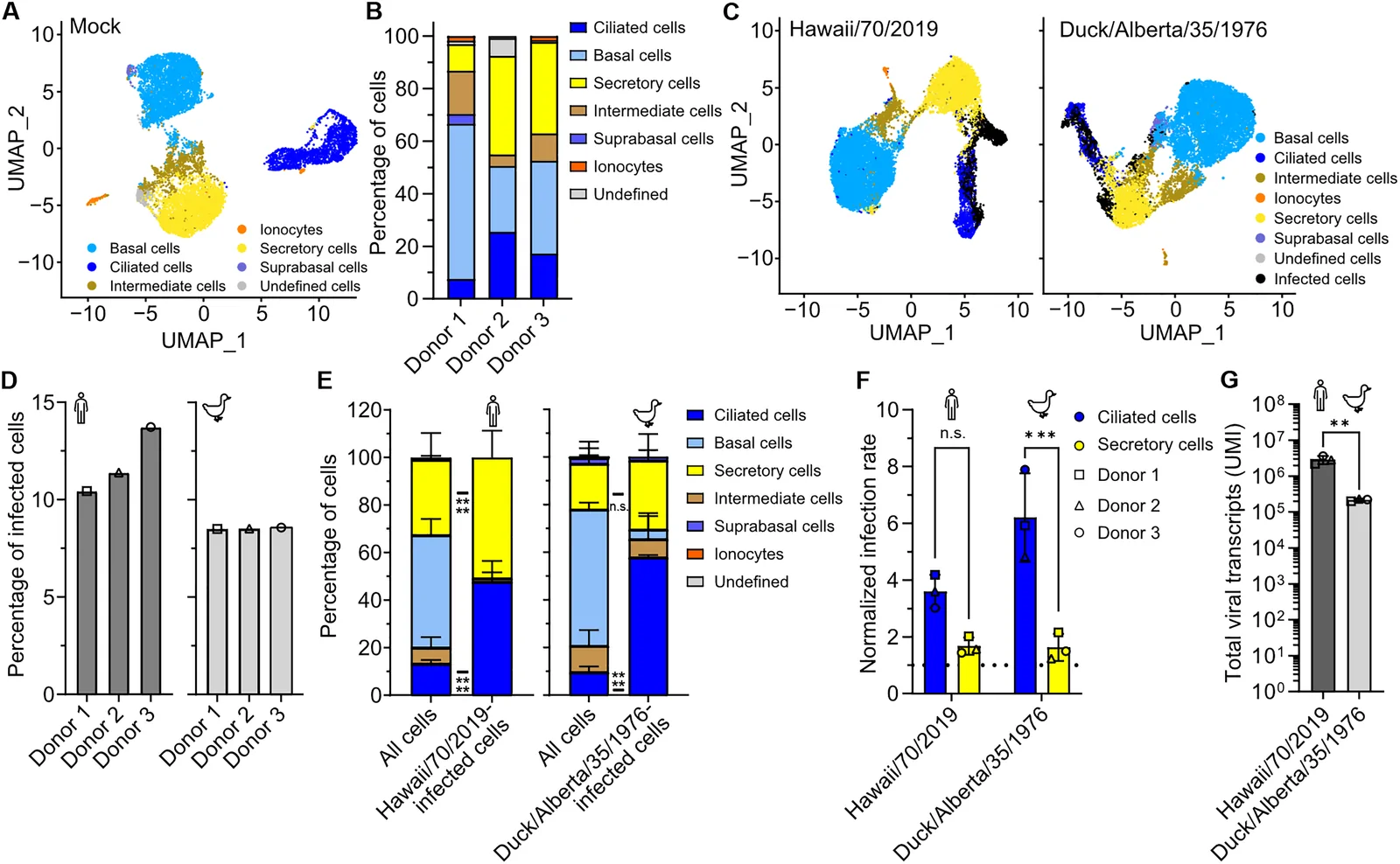
Single-cell RNA transcriptomics reveal divergent bronchial epithelial cell tropism of human and avian IAVs. (**A**) Uniform manifold approximation and projection (UMAP) visualization of single-cell RNA sequencing (scRNA-seq) data from pooled mock-infected BEpCs (donors 1 to 3). Cell-subtype allocation was based on expression of canonical subtype-specific markers: Basal cells (light blue): *TP63*, *KRT5*, *DAPL1*, *KRT15*, *ITGA6*, and *KRT17*; secretory cells (yellow): *SCGB3A1*, *SCGB1A1*, *MUC5B*, *MUC5AC*, *SPDEF*, *TCN1*, *BPIFB1*, *SPRR3*, and *AGR2*; ciliated cells (dark blue): *FOXJ1*, *CAPS*, *TP73*, and *CCDC78*; ionocytes (orange): *CFTR*, *ASCL3*, *FOXI1*, and *ATP6V1C2*; suprabasal cells (violet): *KRT6A* and *KRT15*; intermediate cells (brown): clusters expressing markers of basal and secretory cells; and undefined cells (gray): clusters not assignable to known epithelial subtype. (**B**) Relative cell-type composition of BEpCs of samples in (A). (**C**) UMAP visualization of pooled scRNA-seq data of BEpCs (donors 1 to 3) infected with Hawaii/70/2019 or Duck/Alberta/35/1976 (MOI of 1; 6 hpi). Allocated cell subtypes and infected cell populations (black) are highlighted. Infected cells were defined by the presence of transcripts from at least four of the eight viral genome segments. (**D**) Total percentage of infected cells of samples in (C). (**E**) Relative cell-type compositions of total and infected cell populations from (C). (**F**) Normalized infection rates of samples in (C). Rates represent the percentage of infected ciliated or secretory cells normalized to the total infected cell population. (**G**) Total viral transcripts detected in samples in (C). Unique molecular identifiers (UMI). [(F) and (G)] Data from donors 1 to 3 pooled and distinguished by symbol shape: donor 1 (square), donor 2 (triangle), and donor 3 (circle). [(A) to (G)] Data represent *n* = 1 experiments per donor (donors 1 to 3). [(E) to (G)] Data represent means ± SD. [(E) and (F)] Statistical significance was determined using two-way ANOVA with Šídák’s multiple comparisons test. (G) Statistical significance was determined using an unpaired *t* test. **P ≤ 0.01, ***P ≤ 0.001, and ****P ≤ 0.0001. n.s., not significant.

### Human ANP32 incompatibility underlies reduced IAV RNA levels

In the next step, we aimed to understand the viral and host determinants underpinning the differences in viral mRNA and protein levels between hIAV and aIAV within 6 to 7 hpi in primary human airway cultures (Figs. 1G and 2G). Using reverse transcription quantitative polymerase chain reaction (RT-qPCR) analysis of the viral M segment levels (6 hpi), we confirmed higher viral RNA levels in the hIAV-infected over aIAV-infected samples (Fig. 3A). Using pseudobulk expression profiles of the scRNA-seq data presented in Fig. 2, we performed a pseudobulk differential expression analysis comparing Hawaii/70/2019-infected cells over Duck/Alberta/35/1976-infected cells (6 hpi; Fig. 3B). This comparison only included cells that are defined as infected cells. We found that the main hits overrepresented in aIAV infected cells can be assigned to the antiviral innate immune response (Fig. 3C), with a strong presence of ISGs (Fig. 3B). When analyzing ISG expression on a single-cell level, we also detected higher signals for *MX1*, *ISG15*, *IFIT1*, and *IFI44* (fig. S5, A to C). This approach further revealed that ISG expression was not limited to infected cells but included bystander cells for both samples infected with the human and the avian virus (fig. S5, D and E). Moreover, ISG expression levels per cell did not show a clear correlation, positively or negatively, with their viral RNA levels in ciliated or secretory cells (fig. S5, F to I). The correlation coefficients (*r*) ranged only from 0.037 to 0.35 for Duck/Alberta/35/1976, whereas those for Hawaii/70/2019 ranged from −0.18 to 0.12.

**Fig. 3.**
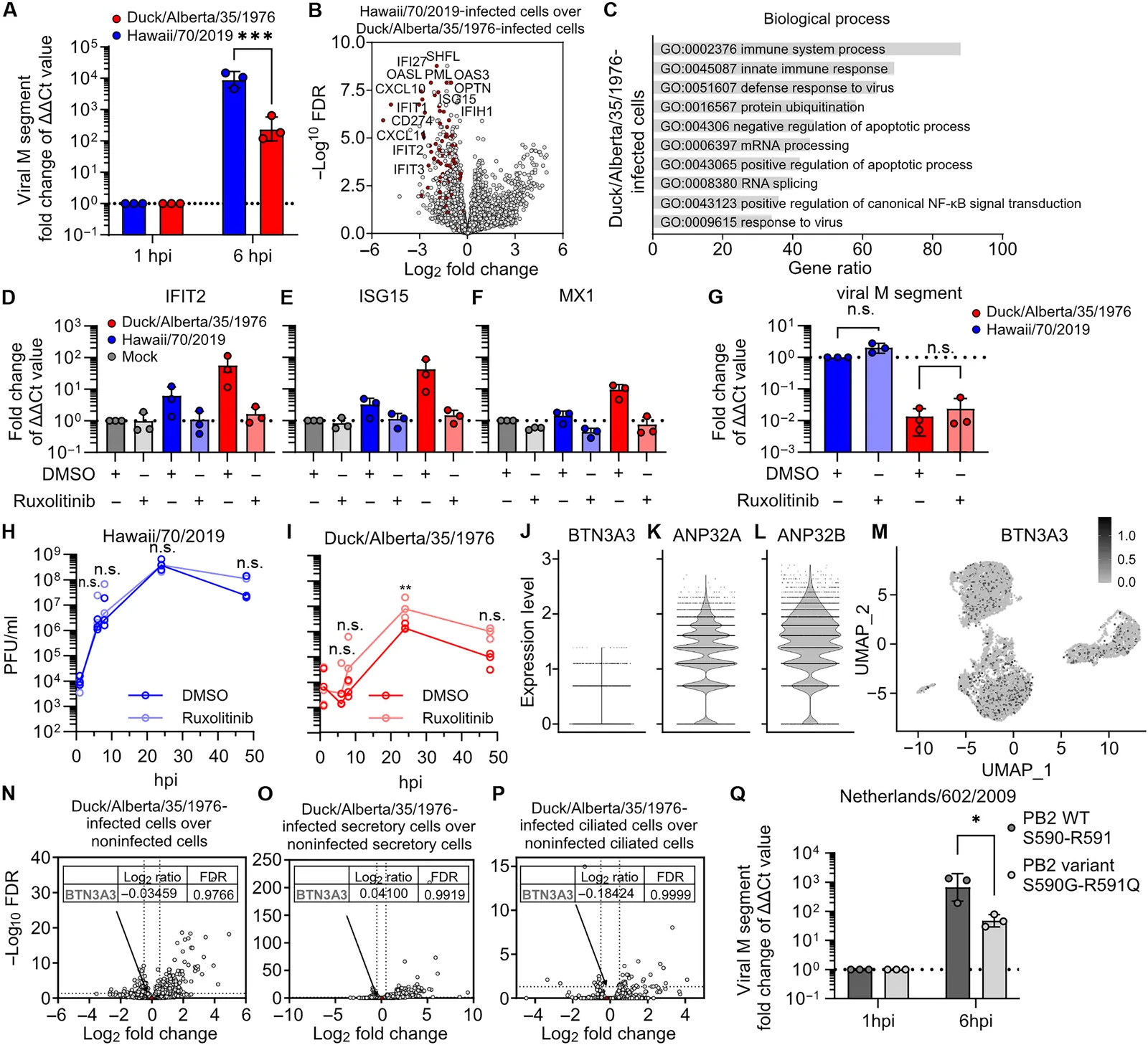
Early IAV replication efficiency depends on ANP32 compatibility. (**A**) BEpCs (donors 1 to 3) were infected with Hawaii/70/2019 or Duck/Alberta/35/1976 (MOI of 1; 1/6 hrs). Enrichment of M segment transcripts relative to 1 hpi. (**B**) scRNA-seq data (Fig. 2C) were pooled to generate pseudobulk expression profiles, and differential expression analysis was performed of Hawaii/70/2019-infected over Duck/Alberta/35/1976-infected cells. Genes from GO:0045087 highlighted (red). (**C**) Hypergeometric overrepresentation analysis of differentially expressed genes in (B); top 10 biological processes, ranked with number of genes associated, are shown. Threshold: *P ≤ 0.01. NF-κB, nuclear factor κB. (**D** to **G**) BEpCs (donor 2) were pretreated with DMSO or ruxolitinib for 86 hours before infection with mock, Hawaii/70/2019, or Duck/Alberta/35/1976 (MOI of 1; 6 hpi). Enrichment of transcript levels of *IFIT2*, *ISG15*, *MX1*, and viral M segment presented as fold change relative to (D) to (F) mock DMSO or (G) Hawaii/70/2019 DMSO. (**H** and **I**) BEpCs (donor 2) were pretreated and infected as in (D). At indicated times, viral release was determined. (**J** to **L**) Single-cell transform (SCTransform)–normalized expression of (J) *BTN3A3*, (K) *ANP32A*, and (L) *ANP32B* within cells from mock-infected cells (donors pooled). (**M**) UMAP visualization of BTN3A3 SCTransform-normalized expression within mock-infected cells (donors pooled). (**N** to **P**) Differential expression analysis of Duck/Alberta/36/1976 (N) infected over noninfected cells, (O) infected secretory over noninfected secretory cells, and (P) infected ciliated over noninfected ciliated cells. *BTN3A3* highlighted (dark red). (**Q**) BEpCs (donor 2) were infected with Netherlands/602/2009 PB2 S590-R591 or S590G-R591Q (MOI of 0.6). At 1 and 6 hpi, RNA was analyzed by RT-qPCR. [(A) to (C) and (J) to (P)] Data represent *n* = 1 experiments per donor (means ± SD). [(D) to (I) and (Q)] Data represent means ± SD in (D) to (G) and (Q) or medians in (H) and (I) from *n* = 3 independent experiments. [(A), (G) to (I), and (Q)] Statistical significance determined using two-way ANOVA with Šídák’s multiple comparisons test. *P ≤ 0.05, **P ≤ 0.01, and ***P ≤ 0.001. n.s., not significant.

To test for a potential link between higher ISG expression and decreased viral RNA/protein levels in aIAV-infected cells, we inhibited the interferon-stimulated pathway and examined its effects on viral replication. We pretreated BEpCs with ruxolitinib, a Janus kinase inhibitor, or control, before infecting with Hawaii/70/2019 or Duck/Alberta/35/1976. As validated by the transcript levels of *IFIT2*, *ISG15*, *MX1*, and *IFIT1*, we successfully inhibited the up-regulation of these ISGs at 6 hpi (Fig. 3, D to F, and fig. S6, A to C). Yet, the viral M segment levels of neither IAV were significantly affected by the presence of ruxolitinib (Fig. 3G). Of note, IFIT1, IFIT2, and ISG15 can also be directly induced by interferon regulatory factor–3 activation independent of interferons but were still strongly down-regulated upon ruxolitinib treatment. To test whether the presence of ruxolitinib affects viral growth in multicycle replication, we harvested apically released viruses from these cultures between 1 and 48 hpi (Fig. 3, H and I) and observed a significant positive effect on avian viral release (at 24 hpi). These results combined highlight that, while the avian virus already leads to a stronger ISG response during early BEpC infection, this does not account for the difference in viral replication at 6 hpi. However, the interferon response affects viral growth in subsequent infection rounds.

We next aimed to assess the role of known human host factors that can either promote (hANP32A and hANP32B) (*12*) or inhibit (BTN3A3) (*17*) IAV replication, with differential effects on human versus avian IAVs. The human IAVs used in this study code for 627 K or the SR polymorphism at amino acid positions 590 and 591 in PB2 and can therefore use hANP32 proteins to replicate their genome efficiently (*29*, *30*). They also code for NP 313 V or 313Y and thus can escape BTN3A3 restriction (fig. S6D) (*17*). On the other hand, avian viruses used here do not carry substitutions known to enable hANP32 usage or BTN3A3 escape (fig. S6D). Transcript analysis of the mock-infected cultures confirmed the expression of the three genes (Fig. 3, J to L). The *ANP32* transcripts showed high expression across the cell population, whereas *BTN3A3* showed a comparably low expression profile (fig. S6, E to J). As *BTN3A3* expression was restricted to a subset of cells (Fig. 3M), we hypothesized that *BTN3A3* expression would be overrepresented in noninfected cells if it was responsible for the observed difference of aIAV RNA levels. However, no preference for infection of *BTN3A3*-negative cells was detected, even upon restriction of the analysis to a specific cell-subtype population (ciliated or secretory cells) (Fig. 3, N to P), suggesting that BTN3A3 is not the root of the difference in early viral RNA levels between hIAVs and aIAVs in the primary human airway cultures. Next, to determine whether the viral polymerase adaptation to hANP32 usage underlies the differences in viral replication efficiency, we generated a recombinant H1N1 virus based on the Netherlands/602/2009 strain in which the PB2 segment was mutated to encode the hANP32-maladapted S590G-R591Q sequence (fig. S6D). Using RT-qPCR analysis, we found that the loss of the PB2 SR polymorphism significantly impaired replication efficiency at 6 hpi (Fig. 3Q), highlighting the importance of the host factor ANP32 for early viral replication.

### Virus tropism is linked to HA and sialic acid distribution

To determine the basis of the cellular tropism of A/Hawaii/70/2019 and A/Duck/Alberta/35/1976, we first examined viral determinants and generated recombinant 7+1 viruses. These viruses carry the internal segments (PB2, PB1, PA, NP, M, and NS) of the laboratory-adapted H1N1 strain A/WSN/1933 and express either the envelope protein HA or neuraminidase (NA) of either Hawaii/70/2019 or Duck/Alberta/35/1976 (Fig. 4A and fig. S7A). At 7 hpi, the parental strain A/WSN/1933 showed no preference for infecting either ciliated or apical nonciliated cells. Likewise, exchanging the NA did not alter tropism. However, substitution of HA for the avian variant was sufficient to induce a clear and significant preference for ciliated over nonciliated bronchial epithelial cells. These infection patterns directly correlate with the tropism seen in the flow cytometry (Fig. 1E), microscopy (Fig. 1F), and scRNA-seq (Fig. 2F) data. Given that HA mediates viral attachment and entry via sialic acid residues on the cell surface, we examined the distribution of α2,3- or α2,6-linked sialic acids on the whole surface of BEpCs (pan-membrane staining). Using the flow cytometry panel for the different cell types together with lectins for either α2,3- or α2,6-linked sialic acids (Fig. 4, B to G; and figs. S7B and S8), we found that the percentages of α2,3- or α2,6-linked sialic acid–positive cells did not significantly differ between ciliated and secretory cells (Fig. 4, B and E; and fig. S8, A to C and J to L; BEpCs from donors 1 to 3). Likewise, the median fluorescence intensity (MFI) of these stainings, when considering all ciliated and secretory cells, did not differ significantly between the populations (Fig. 4, C and F; and fig. S8, D to F and M to O). However, when analyzing the signal intensity specifically on sialic acid–positive cells (Fig. 4, D and G; fig. S8, G to I and P to R), we found that the α2,3-linked sialic acid signal was consistently higher on ciliated versus secretory cells (Fig. 4D), giving a first indication that ciliated cells display a higher surface density of α2,3-linked sialic acids than α2,3-linked sialic acid–positive secretory cells.

**Fig. 4.**
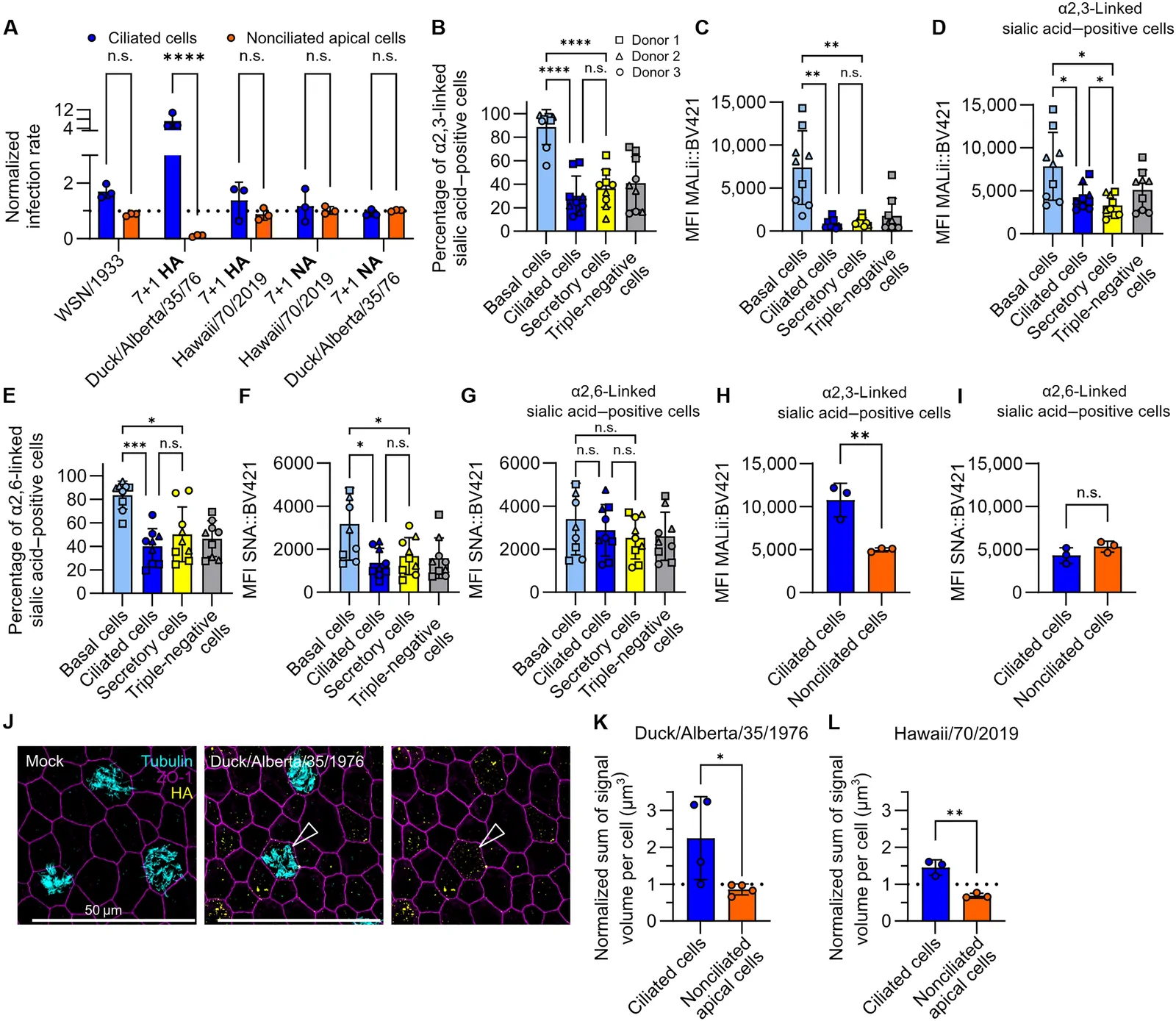
Cell tropism of IAV is linked to apical distribution of sialic acid. (**A**) BEpCs (donor 2) were infected with WSN/1933 or recombinant 7+1 viruses containing seven segments from WSN/1933 strain and HA/NA of Hawaii/70/2019 or Duck/Alberta/35/1976 (7 hpi). Cells were stained for NP, ciliated cells, tight junctions, and nuclei. Percentage of NP positive relative to all cells normalized to total infected cells. (**B** to **G**) BEpCs (donors 1 to 3) were stained for cell surface markers identifying ciliated, basal, secretory cells, and α2,3- and α2,6-linked sialic acids (SA). One repeat was previously published (*45*). [(B) and (E)] Percentage of α2,3- and α2,6-linked SA-positive cells. [(C) and (F)] MFI of α2,3- and α2,6-linked SA. (D and G) MFI of α2,3- and α2,6-linked SA in SA-positive cells. Donors 1 to 3 pooled: donor 1 (square), donor 2 (triangle), and donor 3 (circle). (**H** and **I**) BEpCs (donor 2) were stained for apical (H) α2,3- and (I) α2,6-linked SA and ciliated cells. MFI of apical SA staining on SA-positive cells. (**J** to **L**) BEpCs (donor 2) were precooled on ice and infected with (K) Duck/Alberta/35/1976 or (L) Hawaii/70/2019 for 1 hour on ice. Cells were stained for HA, ciliated cells, tight junctions, and nuclei. Summed viral signal on ciliated and nonciliated apical cells normalized to viral signal present on all cells. (J) Representative images of mock- and Duck/Alberta/35/1976-treated sample. White arrowheads highlight viral HA. [(A) and (H) to (L)] Data represent means ± SD from *n* ≥ 3 independent experiments. [(B) to (G)] Data represent means ± SD from *n* = 3 independent experiments per donor (donors 1 to 3). [(A), (H) to (I), and (K) to (L)] Data represent means ± SD from n ≥ 3 independent experiments. Statistical significance was determined in (A) using two-way ANOVA with Šídák’s multiple comparisons test, in (B) to (G) using RM one-way ANOVA with Tukey’s multiple comparisons test, and in (H) to (I) and (K) to (L) using unpaired *t* test. *P ≤ 0.05, **P ≤ 0.01, ***P ≤ 0.001, and ****P ≤ 0.0001. n.s., not significant.

As the viruses are restricted to the apical surface of the cells for the initial binding and entry, we confined further staining to the apical surface only. Microscopy analysis confirmed the presence of both α2,3- and α2,6-linked sialic acids on the apical surface of ciliated and nonciliated cells (fig. S9A). Furthermore, flow cytometry enabled quantitative assessment of their levels (Fig. 4, H and I; and fig. S9B). While the MFI was comparable between α2,6-linked sialic acid–positive ciliated and nonciliated cells, the α2,3-linked sialic acids were detected at significantly higher levels on ciliated cells versus nonciliated cells. These data highlight that, although levels of α2,3-linked sialic acid on the whole-cell surface barely differed between ciliated and secretory cells, we identified a clear disparity on the apical cell surface. To further delineate the origin of the cellular tropism, we next assessed viral binding to the airway cultures. The viruses were allowed to attach to the apical cell surface of the bronchial human airway cells on ice, after which the viral particles were stained for microscopy analysis (Fig. 4, J to L). Notably, the sum of the viral signal per cell was significantly higher on ciliated than on nonciliated apical cells for both viruses (Fig. 4, K to L). For the avian virus, this is in line with our finding of higher α2,3-linked sialic acid levels on ciliated cells. For the human virus, the binding pattern does not correlate with the α2,6-linked sialic acid levels, suggesting that glycan characteristics beyond linkage and/or additional attachment receptors affect virus binding.

### Preference for sLe^x^ correlates with ciliated cell tropism of avian H1N1

To determine whether glycan structures besides the terminal linkage of the sialic acid residue play a role in the binding efficiency of the viruses, we conducted glycan array analysis for Hawaii/70/2019 and Duck/Alberta/35/1976 (Fig. 5A and fig. S10, A to D) (*31*, *32*). As expected, the avian strain showed a clear preference for α2,3-linked sialic acids (Fig. 5B). Beyond the linkage, we were able to reveal a preference of the aIAV for longer and more branched glycans (Fig. 5B and fig. S10A) and a preference for sialylation of specific glycan arms over others (Fig. 5B and fig. S10B). Specifically, the avian virus prefers sialylation on the mannosyl-glycoprotein *N*-acetylglucosaminyl transferase 4 (MGAT4) over the MGAT5 antenna. Last, aIAV displayed a clear preference for sialyl Lewis X (sLe^x^) epitopes over nonfucosylated α2,3-linked sialic acid residues (Fig. 5B and fig. S10C). Via microscopy and anti-sLe^x^ antibodies, we could confirm the presence of sLe^x^ on the apical surface of ciliated and nonciliated cells (Fig. 5C). Further, via flow cytometry, we quantified the signal of apical sLe^x^ and identified three times higher levels on ciliated versus nonciliated cells (Fig. 5D and fig. S10, E and F), highlighting that the preference of the avian H1N1 strain for sLe^x^ correlates with the glycan presence on the apical epithelium surface. The human IAV, on the other hand, showed, next to the preference for α2,6-linked sialic acids (Fig. 5E), a preference for two *N*-acetyllactosamine (LacNAc) repeats on the sialylated branch over one or three repeats (Fig. 5, F and G), when both branches of a biantennary N-glycan are sialylated. In addition, the human virus also bound biantennary glycans where one branch had three LacNAc repeats and the other branch only one LacNAc repeat (Fig. 5G and fig. S10D). From these glycan array results combined with the viral binding data, we speculate that ciliated cells display more of the biantennary glycans that are preferred by the human virus, while overall α2,6-linked sialic acid levels are comparable between ciliated and secretory cells.

**Fig. 5.**
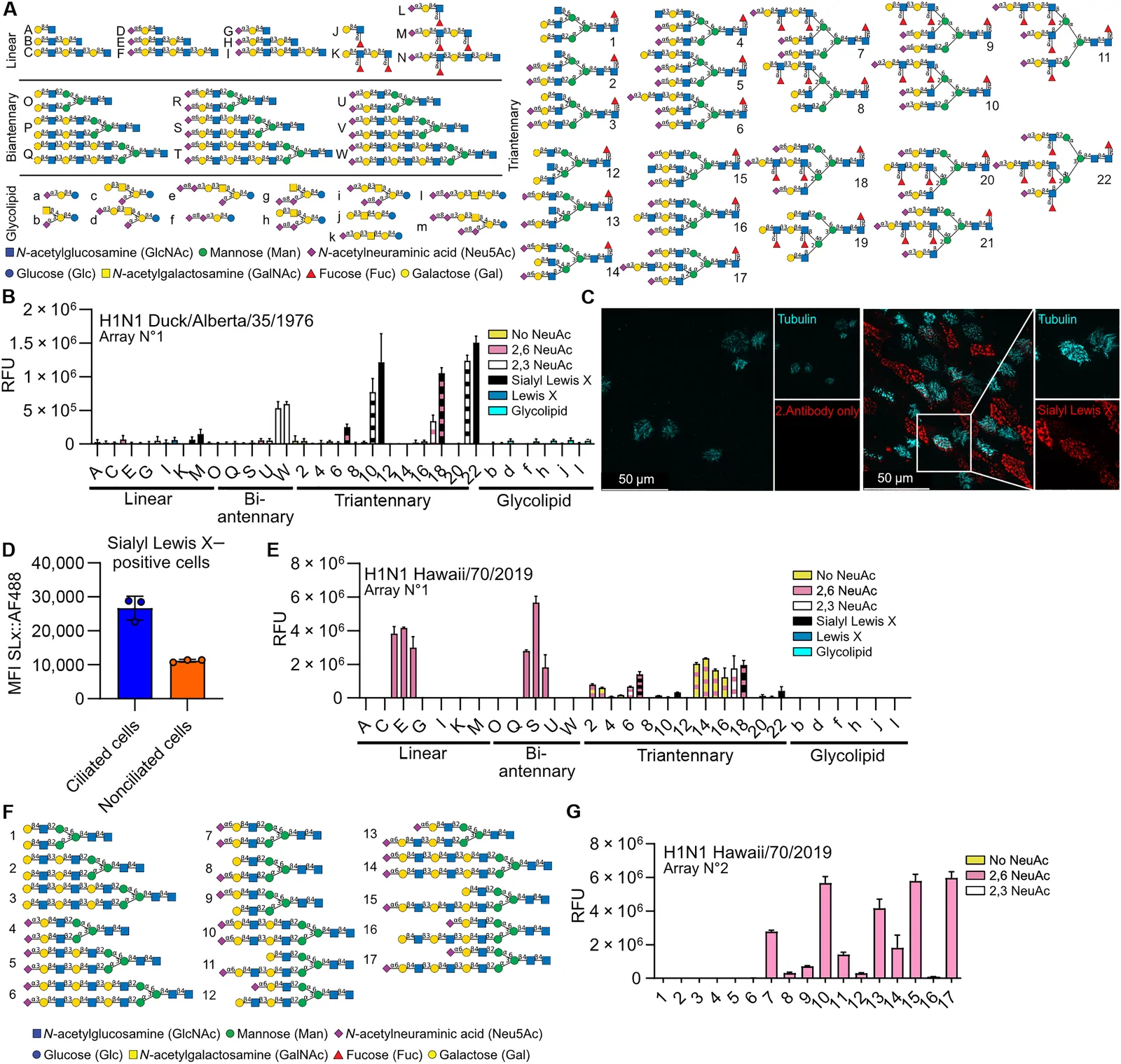
Avian H1N1 preference for sialyl Lewis X correlates with glycan abundance on ciliated cells. (**A**) Synthetic glycans on the microarray N°1 with linear (A to N), biantennary (O to W), triantennary structures (1 to 22), and glycolipids (a to m). (**B**) Microarray N°1 binding of H1N1 A/Duck/Alberta/35/1976. The microarray N°1 tested for glycans with no sialylation (yellow), α2,6-linked NeuAc (pink), α2,3-linked NeuAc (white), Lewis X (blue), sialyl Lewis X (black), and glycolipids (cyan). Striped bars indicate glycans terminating in different epitopes on different arms. (**C**) Representative microscopy images of apical sialyl Lewis X staining on BEpCs from donor 2. The cells were stained for ciliated cells (microtubule dye) and surface sialyl Lewis X. (**D**) BEpCs from donor 2 were stained before trypsinization for ciliated cells (microtubule dye), as well as for apical sialyl Lewis X (SLx). The cells were analyzed by flow cytometry, cellular doublets were excluded by size, and dead cells with a cell viability marker. Shown is the MFI of the apical sialyl Lewis X staining on ciliated versus nonciliated cells, pregated for sialyl Lewis X–positive cells. (**E**) Microarray N°1 binding of A/Hawaii/70/2019. (**F**) Synthetic glycans on the microarray N°2. (**G**) Microarray N°2 binding of H1N1 A/Hawaii/70/2019. The microarray N°2 tested for glycans with no sialylation (yellow), α2,6-linked NeuAc (pink), α2,3-linked NeuAc (white). In (B), (E), and (G), mean relative fluorescence units (RFU) ± SD from *n* = 4 technical repeats are shown. In (D), data represent means ± SD from *n* = 3 repeats.

### Viral replication levels of human H1N1 are cell-subtype specific

Given the significant preference for ciliated cells in binding but not overall infection rate for the human strain, we aimed to test for differences in transcription/replication levels between ciliated and secretory cells. First, we used our scRNA-seq analysis (Fig. 2) and quantified viral mRNA copies in both cell types. Viral mRNA levels were significantly higher in infected secretory versus infected ciliated cells for Hawaii/70/2019 but not for Duck/Alberta/35/1976 (Fig. 6A). In parallel, we also aimed to measure viral NP protein levels in ciliated and secretory cells. While staining of viral NP was not compatible with costaining for the secretory cell marker, we could costain for the ciliated cell marker and thus determine NP levels for nonciliated versus ciliated cells. In line with the transcriptomic data, we detected significantly higher NP levels in the nonciliated cells for the Hawaii/70/2019-infected but not for the Duck/Alberta/35/1976-infected cells (Fig. 6B). Given that the vast majority of infected cells were found to be ciliated or secretory cells (Figs. 1D and 2E), we assume that most of infected nonciliated cells are secretory cells. Based on these findings, we investigated whether known host factors contribute to the virus- and cell-type–specific differences in replication efficiency. Using pseudobulk expression profiles of the scRNA-seq data, we performed differential expression analysis comparing mock secretory over ciliated cells (Fig. 6C). Gene ontology (GO) analysis of the transcripts enriched in each cell type revealed that ciliated cells were primarily associated with cilium-related processes (Fig. 6D), whereas secretory cells showed an enrichment for pathways related to transcription including positive regulation of RNA polymerase II (GO:0045944) (Fig. 6E). Given that previous work has established that IAV transcription depends on RNA polymerase II activity (*33*–*36*), these findings suggest that viral transcription may be more efficient in secretory cells due to higher active RNA polymerase II activity. To assess whether cell-type–specific, infection-induced transcriptional responses, such as the type I interferon response, may further contribute to the difference in viral replication, we compared the induction of infection-induced genes relative to the mock controls (Fig. 6F). As the top infection-induced transcripts exhibited consistently higher fold changes in secretory cells than ciliated cells, we concluded that the cell-type–specific infection responses, including the ISG expression levels, cannot explain the increase in RNA production observed in secretory cells. Last, the comparison of ANP32 expression revealed that secretory cells expressed ∼25% higher levels of ANP32B and 17% higher levels of ANP32A (Fig. 6, G and H). Using the Netherlands/602/2009 viral pair, we found that the ability to use human ANP32 proteins not only influences overall replication efficiency (Fig. 3Q) but also accounts for the higher viral protein levels observed in secretory compared with ciliated cells (Fig. 6I and fig. S11, A and B). It therefore highlights the importance of the host factor ANP32 in early viral replication and cellular tropism.

**Fig. 6.**
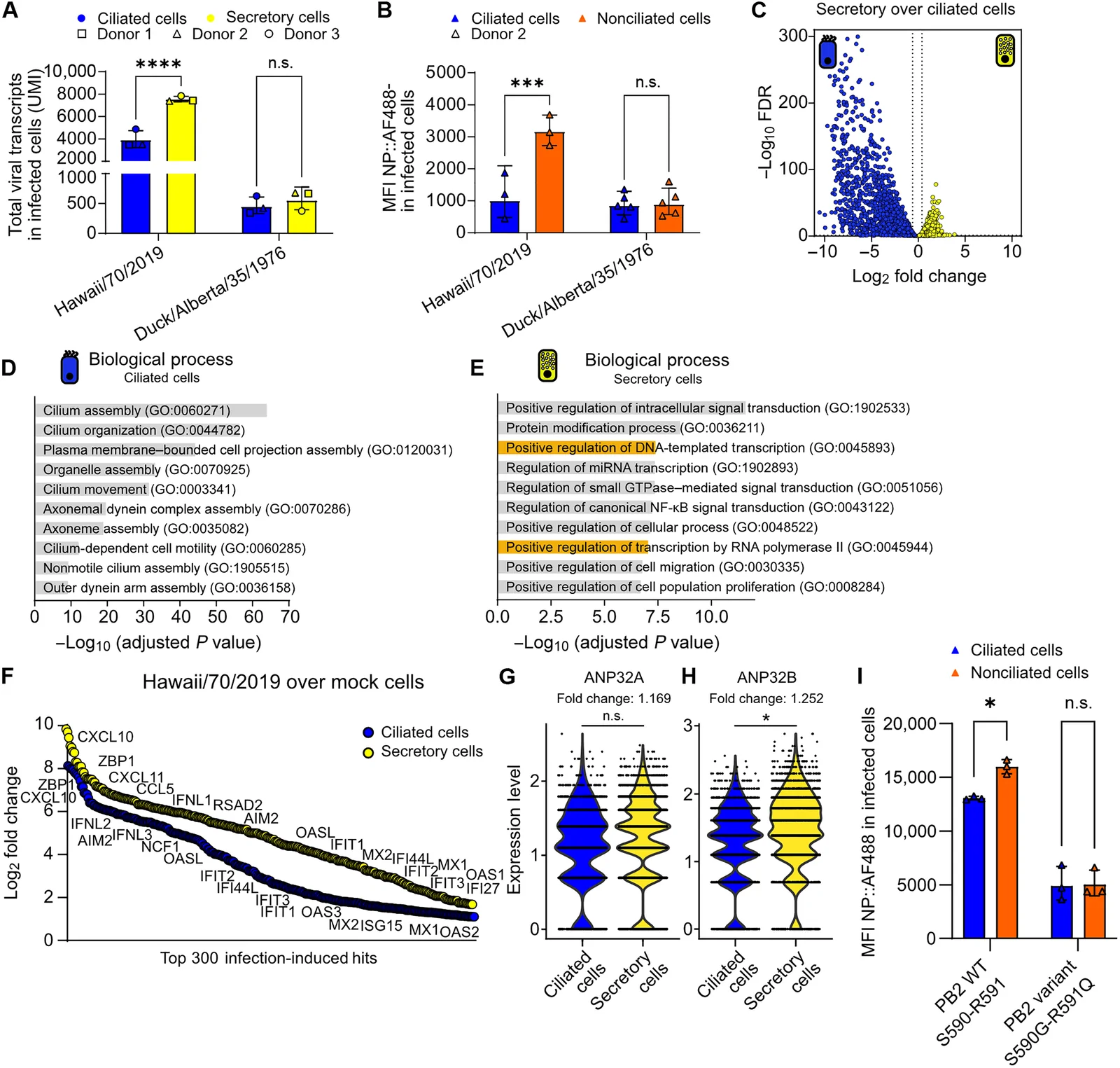
Early viral RNA and protein levels of human H1N1 are cell-subtype specific. (**A**) BEpCs were infected with H1N1 Hawaii/70/2019 or Duck/Alberta/35/1976 (MOI of 1; 6 hpi), analyzed by scRNA-seq, as in Fig. 2C. Total UMIs detected in infected secretory and ciliated cells. Data from donors 1 to 3 pooled: donor 1 (square), donor 2 (triangle), and donor 3 (circle). (**B**) BEpCs from donor 2 were infected with Hawaii/70/2019 or Duck/Alberta/35/1976 (MOI of 5; 7 hpi). Cells were stained for NP and ciliated cells. MFI of NP in infected ciliated and nonciliated cells was quantified. (**C**) scRNA-seq mock data were pooled to generate pseudobulk expression profiles, and differential expression analysis of mock secretory cells over mock ciliated cells was performed. (**D** and **E**) Gene ontology (GO) analysis of differentially expressed genes (DEGs) from (C). Plot shows top 10 enriched biological processes, ranked by adjusted *P* value for (D) ciliated cell and (E) secretory cell population. Threshold for DEG: *P ≤ 0.05, log_2_ fold change of 0.5. (**F**) scRNA-seq data were pooled to generate pseudobulk expression profiles, and differential expression analysis of Hawaii/70/2019-infected samples over mock samples was performed for ciliated and secretory cells, separately. (**G** and **H**) Single-cell transform–normalized expression of (G) *ANP32A* and (H) *ANP32B* within ciliated and secretory cells of the scRNA-seq dataset from mock-infected cells. (**I**) BEpCs from donor 2 were infected with Netherlands/602/2009 PB2 S590-R591 or PB2 S590G-R591Q (MOI of 0.45). At 8 hpi, cells were stained for NP and ciliated cells. MFI of NP in infected ciliated and nonciliated cells was quantified. [(A) and (C) to (H)] Data represent *n* = 1 experiment per donor (donors 1 to 3). [(B) and (I)] Data represent *n* ≥ 3 independent experiments. [(A), (B), and (I)] Data represent means ± SD, statistical significance determined using two-way ANOVA with Šídák’s multiple comparisons test. *P ≤ 0.05, ***P ≤ 0.001, and ****P ≤ 0.0001. n.s., not significant.

In sum, our results show that human and avian IAVs infect ciliated and secretory cells in the human airway epithelium. aIAV displays a strong preference for ciliated cells, which is driven by the higher abundance of sLe^x^ on ciliated apical cell surfaces and thus stronger viral binding. Combined with comparable replication efficiencies in both cell types, aIAV displays an overall preference for ciliated cells. In contrast, the tropism of human IAV is driven by both differential viral binding and differential RNA levels across the two cell types. While binding is stronger on ciliated cells, possibly via higher abundance of biantennary glycans with two LacNac repeats, viral RNA and protein production are more efficient in secretory cells due to differential *ANP32A/B* expression. This leads to an overall dual tropism for both cell types for human IAV.

## DISCUSSION

A zoonotic spillover from the aIAV reservoir into the human host is initiated by a productive infection in the airway epithelium, where early host-pathogen interactions determine the outcome. Our study demonstrates that such infections exhibit distinct tropism patterns compared with those of seasonal human influenza viruses. Our findings highlight that the cellular tropism is not dictated by individual pro- or antiviral factors in isolation but instead arises from the interplay of multiple viral and host determinants acting across different stages of the viral life cycle.

Using a differentiated human airway epithelial model, we provide a comprehensive system to resolve the heterogeneity of IAV infection at the cellular level, enabling direct comparison between human and avian IAV infections as well as stratification by epithelial cell subtypes. Through this approach, we redefine IAV tropism in the human airway and show that at least two key steps in the viral life cycle (viral binding and viral RNA production) shape the outcome with stage-specific differences that are not apparent when only assessing the overall infection.

For viral binding, we show that the overall glycan structure influences it, not solely sialic acid linkages. While it has been reported before that certain aIAV strains preferentially bind sialyl Lewis X (sLe^x^) (H5, H6, H7, and H9) (*31*, *37*, *38*), we now show that this preference may be more widespread, demonstrating preferential binding of an avian H1N1 virus to sLe^x^. Moreover, we uncover that sLe^x^ is enriched on ciliated apical cell surfaces, the primary target of avian viruses in our model. On the other hand, human-adapted H3N2 strains are known to have evolved to bind to extended (≥3 LacNAc repeats) branched glycans (*39*–*43*). Human H1N1 strains have been shown to preferentially bind sialylated glycans with two LacNAc repeats (*31*, *42*, *43*), which is the preference that we have identified for our human H1N1 strain. By combining pan-membrane and apical-surface-specific analyses, we uncover pronounced differences in glycan distribution not only across epithelial cell subtypes but also between staining approaches. Whereas previous studies relied on nonquantitative immunofluorescence (*18*, *22*, *23*) or focused exclusively on pan-membrane staining (*24*), our quantitative approach reveals distinct apical versus pan-membrane patterns of α2,3-linked sialic acids. This provides a more refined, cell-subtype–specific view of the epithelial glycome than previously reported. These findings emphasize that both spatial and cell-subtype–specific glycan presentation can influence viral tropism.

For viral replication, we find that the host factors ANP32A/B represent a major limitation for aIAV within the initial replication round in the primary airway cultures. This highlights the importance of polymerase compatibility as a barrier during early zoonotic infection and is in line with previous reports that studied ANP32A/B function in more conventional cell lines (*12*, *29*, *30*). Additionally, the preferential replication of seasonal human viruses in secretory cells suggests that ciliated cells may represent a suboptimal environment for viral replication and that differential expression of ANP32A/B contributes to this observation. Together, our data indicate that viral entry is not the primary limiting step but that efficient replication represents a key bottleneck in this model.

Last, our findings also provide an important context for previous work. Roach *et al.* (*24*) reported that pandemic 2009 strains produce significantly higher levels of viral transcripts in bronchial secretory cells compared with ciliated cells at 6 hpi. We extend this analysis by identifying the role of ANP32 in this process and by comparing aIAV infections within the same system and reveal that the increased viral RNA levels in secretory cells are specific for human IAV. We therefore underscore fundamental differences in host adaptation.

It is important to note that, while the differentiated primary cell cultures provide a powerful system to study the heterogeneity and complexity of the human airway, they also show limitations. In particular, the current difficulty in genetically manipulating these cultures restricts mechanistic investigations. This is especially apparent when aiming to study cell-subtype–specific aspects. For example, it remains challenging to dissect the correlation between human viral binding preferences and specific N-glycan structures directly in such complex systems.

In summary, our study provides a comprehensive view of IAV tropism that integrates both extracellular attachment and intracellular replication determinants. We show that cellular susceptibility is shaped not only by the glycan landscape but also by cell-type–specific replication capacity, supporting a multifactorial model of early IAV infection and zoonotic transmission. Together, these findings represent a conceptual advance in our understanding of influenza virus host adaptation: By combining a physiologically relevant model with a stage-resolved analysis of infection, this work moves beyond receptor-centric paradigms and identifies intracellular compatibility as a critical determinant of zoonotic success.

## MATERIALS AND METHODS

### Cell lines

Human embryonic kidney (HEK) 293T cells (CRL-3216) and MDCK cells (CCL-34) were purchased from the American Type Culture Collection (ATCC). The cells were cultured at 37°C, 5% CO_2_, and >80% relative humidity in Dulbecco’s modified Eagle’s medium (DMEM; Gibco, no. 41966-052) supplemented with 10% heat-inactivated fetal calf serum (FCS; Gibco, no. A5256701), and penicillin-streptomycin (100 U/ml; Gibco, no. 15140-122).

### Primary human epithelial cultures

Primary human BEpCs were purchased from Epithelix (no. EP51AB). Cells from three independent donors were used: BEpC_AB079 (donor 1; male, 62 years old, Hispanic, nonsmoker), BEpC_AB051 (donor 2; male, 53 years old, Caucasian, nonsmoker), and BEpC_AB0839 (donor 3; male, 59 years old, Caucasian, nonsmoker). Cells were grown in airway epithelium basal growth medium (PromoCell, no. C-21260) supplemented with the airway epithelial cell growth medium supplement pack (PromoCell, no. C-39160) and 10 μM Y-27632 (ROCK inhibitor; Tocris, no. 1254). For cellular differentiation, the transwell plates with 12-mm (Corning, no. CLS3460) or 6.5-mm (Corning, no. CLS3470) filter inserts were coated with collagen. The human collagen type 1 (Sigma-Aldrich, no. C7774) stock in 0.5 M acetic acid (Merck, no. 100063.1000) was diluted to 0.15 mg/ml in phosphate-buffered saline (PBS) before coating the filters. The primary cells were seeded onto the filter inserts in a 1:1 mixture of airway epithelium basal growth medium and DMEM, which was supplemented with airway epithelial cell growth medium supplement pack (Gray’s medium). The cells were grown until confluency was reached. For the cellular differentiation at the ALI, Gray’s medium was removed from the apical side of the filter inserts, and Gray’s medium in the basal compartment was supplemented with retinoic acid (150 ng/ml; Sigma-Aldrich, no. R2625). The cells were cultured at ALI for a minimum of 28 days before use. Medium was refreshed in the basal compartment every 2 to 3 days, and epithelial integrity was monitored weekly by determining the transepithelial electrical resistance using an ERS-2 meter (Millicell, no. MERS00002).

### Viruses

The IAV strains H1N1 A/Duck/Alberta/35/1976, H3N8 A/Duck/Ukraine/1/1963, and H11N6 A/Duck/England/1/1956 were grown in 10-day-old embryonated chicken eggs. H1N1 A/Hawaii/70/2019 was grown in primary human bronchial epithelial cells. H1N1 A/Pennsylvania/02/2021, H3N2 A/Tasmania/503/2020, H3N2 A/Darwin/6/2021, H1N1 A/Netherlands/602/2009, and H1N1 A/WSN/1933 were grown on MDCK cells. Virus stocks were titrated by plaque assay on MDCK cells. Virus sequences were verified by next-generation sequencing.

### Infection of primary human epithelial cells

BEpCs were washed three times (for a total of 15 min at 37°C) with Dulbecco’s PBS (Gibco, no. 14190144) to remove the mucus from the apical side of the inserts. Cells were infected with the indicated viral strain at the indicated MOI in infection PBS [PBSi; PBS supplemented with 0.3% bovine serum albumin (BSA) solution (Sigma-Aldrich, no. A1595), 1 mM Ca^2+^/Mg^2+^, and penicillin-streptomycin (100 U/ml; Gibco, no. 15140-122)]. After incubation for 1 hour at 37°C, the inoculum was removed, and the apical side of the insert was washed once with PBS.

### Microscopy analysis of viral binding on primary human bronchial cultures

BEpCs were washed three times (for a total of 15 min at 37°C) with PBS (Gibco, no. 14190144) to remove the mucus from the apical side of the inserts. Cells were stained for cilia, using the SiR-tubulin kit (1:1000; Cytoskeleton/Spirochrome, no. CY-SC002) for 1 hour at 37°C in Gray’s medium on the apical side of the filter inserts. The cells were washed once with PBS and precooled on ice for 15 min. Viral copies (6.4 × 10^9^) per insert were added apically to the cells on ice. At 1 hour post–viral addition, the unbound virus was washed off, and the cells were fixed with 4% paraformaldehyde (PFA) solution (Electron Microscopy Sciences, no. 15710) in PBS and blocked for unspecific staining with blocking buffer N°1 [BB1; PBS supplemented with 3% BSA (Sigma-Aldrich, no. A7906)] for 30 min at room temperature (RT). The viral particles were stained using an anti-HA 1.12 antibody (1:100) (*44*) for 1 hour at RT. The unbound antibodies were washed off three times with BB1 before permeabilizing the cells for 15 min with the permeabilization buffer N°1 [PB1; PBS supplemented with 50 mM ammonium chloride (Sigma-Aldrich, no. 254134), 0.1% saponin (Sigma-Aldrich, no. 47036), and 2% BSA (Sigma-Aldrich, no. A7906)]. Next, cells were stained with the primary antibody against tight junctions, anti–ZO-1 (1:50; Thermo Fisher Scientific, no. 61-7300), for 1 hour at RT. The unbound antibodies were washed off three times with PB1, before the cells were stained with the secondary antibodies: Alexa Fluor 546 donkey anti-rabbit immunoglobulin G (IgG) highly cross-adsorbed secondary antibody (1:1000; Invitrogen, no. A10040) and Alexa Fluor 488 goat antihuman IgG Fc recombinant secondary antibody (1:500; Invitrogen, no. A55747). The nuclei were stained with 4′,6-diamidino-2-phénylindole dihydrochloride (DAPI; 1:1000; Roche, no. 10236276001). The cell-containing membranes were mounted on a microscope slide by using ProLong gold antifade mountant (Thermo Fisher Scientific, no. P36930). Cells were imaged using the DMi8 microscope (Leica, Germany) and processed using the THUNDER large volume computational clearing algorithm (Leica). Maximum projection images of *z*-stacks were generated using the LAS X software (Leica). The images were analyzed using Imaris 10.2 (Oxford Instruments) to quantify the viral signal on the surface of ciliated and nonciliated cells. Data are visualized using GraphPad Prism version 11.0.

### Microscopy analysis of infected primary human bronchial cultures

BEpCs were infected with the respective viruses and MOIs as indicated previously. One hour before fixation, the cells were stained for cilia, using the SiR-tubulin kit (1:1000; Cytoskeleton/Spirochrome, no. CY-SC002) for 1 hour at 37°C in Gray’s medium on the apical side of the filter inserts. The cells were washed once with PBS and then fixed with 4% PFA (Electron Microscopy Sciences, no. 15710) in PBS. The samples were permeabilized with permeabilization buffer N°1 [PB1; PBS supplemented with 50 mM ammonium chloride (Sigma-Aldrich, no. 254134), 0.1% saponin (Sigma-Aldrich, no. 47036), and 2% BSA (Sigma-Aldrich, no. A7906)] for 30 min at RT. Next, cells were stained with the primary antibodies: anti-NP (1:5; ATCC, no. HB-65) and anti–ZO-1 (1:50; Thermo Fisher Scientific, no. 61-7300) for 1 hour at RT. When indicated, the cells were additionally stained with an anti-HA 1.12 antibody (1:100) (*44*). The unbound antibodies were washed off three times with PB1, before cells were stained with the secondary antibodies: Alexa Fluor 546 donkey anti-rabbit IgG highly cross-adsorbed secondary antibody (1:1000; Invitrogen, no. A10040), and Alexa Fluor 488 goat anti-mouse IgG highly cross-adsorbed secondary antibody (1:1000; Invitrogen, no. A-11029). The nuclei were stained with DAPI (1:1000; Roche, no. 10236276001). The cell-containing membranes were mounted on a microscope slide by using ProLong gold antifade mountant (Thermo Fisher Scientific, no. P36930). Cells were imaged using the DMi8 microscope (Leica, Germany) and processed using the THUNDER large volume computational clearing algorithm (Leica). Maximum projection images of *z*-stacks were generated using the LAS X software (Leica). A minimum of 1000 total/apical cells were counted per sample. The total percentages of infected cells were quantified for all cells containing an apical surface in the selected images (or, if mentioned, for all nuclei present in the selected images). All apical cells negative for the cilia staining were grouped as “nonciliated cell with an apical surface.” Data are visualized using GraphPad Prism version 11.0.

### Flow cytometry analysis of infected primary human bronchial cultures (cell surface staining)

The flow cytometry protocol was developed on the basis of the protocol from Bonser *et al.* (*27*)and previously described by Karakus *et al.* (*45*). In brief, BEpCs were infected with respective viruses and MOIs as indicated previously. At 1 hour before trypsinization, cells were stained for cilia, using the SiR-tubulin kit (1:1000; Cytoskeleton/Spirochrome, no. CY-SC002) for 1 hour at 37°C in Gray’s medium on the apical side of the filter inserts. Cells were washed once with PBS and trypsinized by adding 0.25% trypsin-EDTA (Gibco, no. 25200-056). Cells were resuspended in warm DMEM/F12 (Gibco, no. 11330-032) supplemented with 5% FCS (Gibco, no. A5256701). The resuspended cells were washed once with PBS (805*g* for 5 min at 4°C), and dead cells were stained using the LIVE/DEAD fixable near-IR dead cell marker (1:1000; Thermo Fisher Scientific, no. L10119) in PBS for 30 min at 4°C. The marker was washed away once in PBS. Subsequently, cells were fixed in 4% PFA (Electron Microscopy Sciences, no. 15710) in PBS and blocked with blocking buffer N°2 [BB2; PBS supplemented with 5% normal goat serum (NGS; Gibco, no. 16210-064)] for ∼15 min at RT. Cells were stained with the following cell surface markers: for secretory cells using BV510 mouse antihuman CD66c antibodies (1:100; BD Biosciences, no. 742684), for basal cells using PE/Cyanine7 antihuman CD271 (NGFR) antibodies (1:50; BioLegend, no. 345110), for α2,3-linked sialic acids using *Maackia amurensis* II lectin (MALii; 1:50) (Vector Laboratories, no. B-1265-1), for α2,6-linked sialic acids using *Sambucus nigra* lectin (SNA; 1:425) (Vector Laboratories, no. B-1305) and Brilliant Violet 421 Streptavidin (1:200; BioLegend, no. 405226), and for infected cells using an anti-HA 1.12 antibody (1:100) (*44*) and PE-conjugated antihuman IgG Fc secondary antibody (1:100; Invitrogen, no. 12-4998-82). Cells were washed and resuspended in fluorescence-activated cell sorting (FACS) buffer [FB; PBS supplemented with 2% BSA (Sigma-Aldrich, no. A7906) and 1 mM EDTA (Thermo Fisher Scientific, no. AM9260G)]. Cells were analyzed either with the BD FACSVerse flow cytometer (BD Biosciences) and the BD FACSuite application or with the BD FACSymphony A1 Cell Analyzer (BD Biosciences) and the BD FACSDiva software. A minimum of 10,000 total events were acquired per sample. Data analysis was done using the FlowJo v.10 software (BD Life Sciences). Data are visualized using GraphPad Prism version 11.0.

### Flow cytometry analysis of infected primary human bronchial cultures (intracellular staining)

BEpCs were infected with respective viruses and MOIs as indicated previously. At 1 hour before trypsinization, the cells were stained for cilia, using the SiR-tubulin kit (1:1000; Cytoskeleton/Spirochrome, no. CY-SC002) for 1 hour at 37°C in Gray’s medium on the apical side of the filter inserts. Cells were washed once with PBS and trypsinized by adding 0.25% trypsin-EDTA (Gibco, no. 25200-056). The cells were resuspended in warm DMEM/F12 (Gibco, no. 11330-032) supplemented with 5% FCS (Gibco, no. A5256701). The resuspended cells were washed once with PBS (805*g* for 5 min at 4°C), before staining dead cells using the LIVE/DEAD fixable near-IR dead cell stain (1:1000; Thermo Fisher Scientific, no. L10119) in PBS for 30 min at 4°C. The marker was washed away once in PBS. Subsequently, cells were fixed in 4% PFA (Electron Microscopy Sciences, no. 15710) in PBS and permeabilized with permeabilization buffer N°2 [PB2; PBS supplemented with 2% BSA (Sigma-Aldrich, no. A7906), 1 mM EDTA (Thermo Fisher Scientific, no. AM9260G), and 0.1% (w/v) saponin (Sigma-Aldrich, no. 47036)]. The infected cells were stained with anti-NP antibodies (1:5; ATCC, no. HB-65) for 1 hour at RT in BB2. The unbound antibodies were washed off twice, and the cells were stained with the Alexa Fluor 488 goat anti-mouse IgG highly cross-adsorbed secondary antibody (1:500; Invitrogen, no. A-11029) for 1 hour at RT in BB2. The cells were washed and resuspended in FB. The cells were analyzed either with the BD FACSVerse flow cytometer (BD Biosciences) and the BD FACSuite software or with the BD FACSymphony A1 Cell Analyzer (BD Biosciences) and the BD FACSDiva software. A minimum of 10,000 total events were acquired per sample. Data analysis was done using the FlowJo v.10 software (BD Life Sciences). Data are visualized using GraphPad Prism version 11.0.

### scRNA-seq processing of infected primary human bronchial cultures

BEpC donors 1 to 3 were infected with H1N1 A/Hawaii/70/2019, H1N1 A/Duck/Alberta/35/1976, or mock for a total of 6 hours at an MOI of 1, as indicated above. The cells were then washed once and trypsinized by adding 0.25% trypsin-EDTA (Gibco, no. 25200-056) for a maximum of 10 min at 37°C and resuspended in warm DMEM/F12 (Gibco, no. 11330-032) supplemented with 5% FCS (Gibco, no. A5256701). Cells were centrifuged at 400*g* for 5 min at 4°C and washed once in cold PBS supplemented with 0.04% BSA (Sigma-Aldrich, no. A7906). Subsequently, cells were resuspended in cold 0.04% BSA in PBS and filtered through a 40-μm Flowmi cell strainer (Merck, no. BAH136800040) to ensure single-cell suspension. Using the Countess II automated cell counter (Thermo Fisher Scientific), a final live cell concentration was measured at 600 to 1200 cells/μl. Next, using the Chromium Controller (10x Genomics) and following the “Chromium Next GEM Single Cell 3′ reagent kits v.3.1 (Dual Index)” protocol (10x Genomics), the 3′ gene expression library was constructed. For sequencing, the libraries were processed on an Illumina NovaSeq X Plus flow cell. The sequencing parameters were set according to 10x Genomics recommendations. The Functional Genomics Centre Zurich performed sequencing and subsequent computational data analysis.

### scRNA-seq SUSHI pipeline

scRNA-seq data analysis was performed using the SUSHI framework (*46*), which encompassed the following steps. A chimeric reference genome was constructed using the R package ezRun (v3.19.1) (*47*) by supplementing the human genome assembly (GRCh38.p13, GENCODE Release 42) (*48*) with the nucleotide sequences of the two IAV strains used in this study: H1N1 A/Hawaii/70/2019 and H1N1 A/Duck/Alberta/35/1976. Sample demultiplexing, alignment of reads against this chimeric reference, and generation of feature-barcode count matrices were performed using 10x Genomics Cell Ranger v7.2.0 (*49*), with pre-mRNA counting enabled (--include-introns = true) to capture unspliced transcripts. Downstream analysis was performed on the resulting count matrices using the R package Seurat v5.1.0 (*50*) under R version 4.4.0 and Bioconductor 3.19. Ambient RNA contamination was estimated and removed using DecontX (celda v1.20.0) (*51*) and SoupX (v1.6.2) (*52*). Doublets were identified and removed using scDblFinder (*53*). Low-quality cells were filtered using a median-absolute-deviation (MAD) approach implemented in the R package scater (*54*): Cells deviating by more than 3 MADs below the median library size or number of detected genes or more than 3 MADs above the median mitochondrial read fraction were excluded. Cells with ribosomal protein gene content exceeding 70% were additionally removed. Genes expressed in fewer than 0.01% of cells were filtered out before downstream analysis. Normalization and variance stabilization of count data were performed using SCTransform v2 (sctransform v0.4.1) (*55*), computed on the top 3000 highly variable genes. Dimensionality reduction was performed by principal components analysis, and the top 20 principal components were retained for downstream steps. Cell clusters were identified using the Louvain algorithm at a resolution of 0.6. Cluster marker genes were determined by Wilcoxon rank sum test, retaining genes with an average log_2_ fold change of ≥0.4, Bonferroni-adjusted *P* value of <0.01, expressed in at least 10% of cells in the tested cluster, and with a minimum difference in detection rate of 10% between the tested cluster and all other cells. Cell-subtype allocation was based on the expression of the canonical subtype-specific markers, with a minimum of three canonical markers (two for suprabasal cells) required for cluster allocation. Basal cells: TP63, KRT5, DAPL1, KRT15, ITGA6, and KRT17; secretory cells: SCGB3A1, SCGB1A1, MUC5B, MUC5AC, SPDEF, TCN1, BPIFB1, SPRR3, and AGR2; ciliated cells: FOXJ1, CAPS, TP73, and CCDC78; ionocytes: CFTR, ASCL3, FOXI1, and ATP6V1C2; suprabasal cells: KRT6A and KRT15; intermediate cells: clusters expressing markers of basal and secretory cells; and undefined cells: clusters not assignable to known epithelial subtype. Donor-level batch correction and integration were performed separately for each experimental condition (mock, Hawaii/70/2019 infected, and Duck/Alberta/35/1976 infected) using Harmony (*56*) as implemented within the SUSHI ScSeuratCombine workflow, combining the three donors within each condition. Infected cells were defined as those expressing a minimum of four viral gene segments, where a segment was considered expressed if its unique molecular identifier (UMI) count exceeded 1.5 times the mean expression of that segment across all cells in the same sample. Per-cell viral transcript abundance was computed as log_10_(1 + summed viral UMIs) and summarized per cell type as the mean of these per-cell values within each group. Data are visualized using GraphPad Prism version 11.0 and R 4.6 with Seurat v5.

### Pseudobulk dataset

Pseudobulk and differential expression analysis was performed using the SUSHI framework (*46*). Pseudobulk count matrices were generated by summing raw UMI counts per cell type per sample from the individually processed, cell-type–labeled per-sample Seurat objects, treating each sample as a biological replicate. Differential expression between conditions was tested using DESeq2 (*57*) as implemented in the SUSHI twoGroups workflow, under R version 4.4.0 and Bioconductor 3.19. GO enrichment was performed using the clusterProfiler Bioconductor R package (*58*) against the GO biological process, molecular function, and cellular component ontologies. Overrepresentation analysis was applied to genes reaching a DESeq2 *P* value of ≤0.05 and absolute log_2_ fold change of >0.5; significant GO terms were defined at FDR of ≤0.05. Data are visualized using GraphPad Prism version 11.0 and R 4.6 with Seurat v5.

### Generation of recombinant viruses

To generate recombinant IAVs, the viral genes coding for HA and NA from Hawaii/70/2019 and Duck/Alberta/35/1976 were cloned into ambisense expression plasmids (pDZ), as previously described (*45*). In brief, viral RNA was extracted from the virus stock using the QIAamp Viral RNA mini kit (QIAGEN, no. 52904) according to the manufacturer’s protocol. The viral segments were amplified by PCR with the Q5 High-Fidelity PCR kit (NEB, no. E0555S) using universal primers (table S1) and cloned into the expression vector pDZ using the restriction enzyme Sap I (NEB, no. R0569S). XL10-Gold ultracompetent cells were used for transformation (Agilent, no. 200315). The reverse genetics plasmids for WSN/1933 were a gift from B. G. Hale (Universität Zürich, Switzerland). The reverse genetics system for Netherlands/602/2009 was previously described by Medina *et al.* (*59*). For the mutation of the PB2 plasmid of the Netherlands/602/2009 strain, the segment was amplified with the targeted mutation using designed primer pairs (table S1) and cloned into the pDZ plasmid via In-Fusion cloning (Takara Bio, no. 638948). Stellar competent cells were used for transformation (Thermo Fisher Scientific, no. NC9905817).

The recombinant viruses were rescued, as previously described (*60*). In brief, HEK-293T cells were transfected with 0.5 μg of the following eight plasmids: PB2, PB1, PA, HA, NP, NA, M, and NS-encoding segments of the respective viruses using FuGENE HD transfection reagent (Promega, no. E2312) in OPTI-iMEM (Gibco, no. 31985062). At 24 hours posttransfection, MDCK cells [or MDCK expressing chicken ANP32; (*61*)] were coseeded with TPCK-trypsin (1 μg/ml; Sigma-Aldrich, no. T1426) for additional 48 hours. The rescued viruses were plaque purified and further propagated in MDCK cells (or MDCK expressing chicken ANP32A). Viral sequences were verified by next-generation sequencing, and viral titers were determined by plaque assay on MDCK cells.

### Apical surface glycan staining for immunofluorescence

Mucus was removed by washing with PBS. For the sialidase treatment, cells were preincubated at 37°C for 1 hour with NA (400 mU/ml) from *Vibrio cholerae* (Sigma-Aldrich, no. N6514-1UN). For cilia staining, cells were incubated with the SiR-tubulin kit (1:1000; Cytoskeleton/Spirochrome, no. CY-SC002) for 1 hour at 37°C in Gray’s medium on the apical side of the filter inserts. Cells were fixed with 4% PFA (Electron Microscopy Sciences, no. 15710) in PBS and blocked for unspecific staining with BB1. The apical surface α2,3-linked and α2,6-linked sialic acid linkages were stained using biotinylated MALii (1:50) (Vector Laboratories, no. B-1265-1) or SNA (1:425) (Vector Laboratories, no. B-1305) in BB1 for 1 hour at RT. The apical surface sialyl Lewis X (sLe^x^; 1:100) were stained [BD Biosciences, no. 551344 (clone CSLEX1)] in BB1 for 1 hour at RT.

The lectins/antibodies were washed off three times with BB1 before permeabilizing the cells for 15 min with PB1. For tight junction staining, cells were incubated with an anti–ZO-1 antibody (1:50; Thermo Fisher Scientific, no. 61-7300). For cilia staining in combination with sialic acid staining, an anti-beta IV tubulin antibody (1:400; Abcam, no. ab11315) was used in PB1 for 1 hour at RT. The antibodies were washed away three times with PB1. Last, cells were incubated with secondary proteins/antibodies for 1 hour at RT: streptavidin Alexa Fluor 647 conjugate (1:500; Invitrogen, no. S21374), Alexa Fluor 546 donkey anti-rabbit IgG highly cross-adsorbed secondary antibody (1:1000; Invitrogen, no. A10040), Alexa Fluor 488 goat anti-mouse IgG highly cross-adsorbed secondary antibody, (1:1000; Invitrogen, no. A-11029), and Alexa Fluor 488 goat anti-mouse IgM cross-adsorbed secondary antibody (1:500; Thermo Fisher Scientific, no. A-21042). Nuclei were stained with DAPI (1:1000; Roche, no. 10236276001). The cell-containing membranes were mounted on a microscope slide by using ProLong gold antifade mountant (Thermo Fisher Scientific, no. P36930). The cells were imaged using the DMi8 microscope (Leica, Germany) and processed using the THUNDER large volume computational clearing algorithm (Leica). Maximum projection images of *z*-stacks were generated using the LAS X software (Leica).

### Apical surface glycan staining for flow cytometry

Mucus was removed from the bronchial donor 2 cells. The cells were stained for cilia, using the SiR-tubulin kit (1:1000; Cytoskeleton/Spirochrome, no. CY-SC002) for 1 hour at 37°C in Gray’s medium on the apical side of the filter inserts. The cells were precooled on ice and blocked for unspecific binding on the apical surface with BB2 on ice. The apical surface of the live cells were stained as described for the immunofluorescent analysis: for α2,3-linked sialic acids using MALii (1:50; Vector Laboratories, no. B-1265) and α2,6-linked sialic acid using SNA (1:425; Vector Laboratories, no. B-1305) or for sialyl Lewis X [1:100; BD Biosciences, no. 551344 (clone CSLEX1)] in BB2 for 1 hour on ice. The unbound lectins/antibodies were washed off three times with BB2. Next, cells were stained with Brilliant Violet 421 streptavidin (1:200; BioLegend, no. 405226) or with Alexa Fluor 488 goat anti-mouse IgM cross-adsorbed secondary antibody (1:500; Thermo Fisher Scientific, no. A-21042) for 30 to 60 min on ice. The unbound proteins were washed off three times with BB2 and once with PBS on the apical and the basolateral side. Cells were trypsinized by adding 0.25% trypsin-EDTA (Gibco, no. 25200-056) and resuspended in warm DMEM/F12 (Gibco, no. 11330-032) supplemented with 5% FCS (Gibco, no. A5256701). Cells were then washed once with PBS (805*g* for 5 min at 4°C) before staining the dead cells using the LIVE/DEAD fixable near-IR dead cell stain (1:1000; Thermo Fisher Scientific, no. L10119) in PBS for 30 min at 4°C. The marker was washed away once in PBS. The cells were acquired with the BD FACSymphony A1 Cell Analyzer (BD Biosciences), the data were collected with the FACSDiva software. A minimum of 10,000 total events were acquired per sample. Data analysis was done using the FlowJo v.10 software (BD Life Sciences). Data are visualized using GraphPad Prism version 11.0.

### RNA extraction and RT-qPCR

RNA extraction was conducted using the RNeasy Kit (QIAGEN, no. 74181) and the QIAshredder (QIAGEN, no. 79656) according to the manufacturer’s instructions. Reverse transcription was done using the SuperScript IV reverse transcriptase (Thermo Fisher Scientific, no. 18090050), following the manufacturer’s protocol using random primers (Promega AG, no. C1181), dNTP mix (Invitrogen, no. 18427088), and RNasin Ribonuclease Inhibitor (Promega AG, no. N2615). The qPCR was done using PowerTrack SYBR Green master mix (Thermo Fisher Scientific, no. A46109) using the appropriate primers (table S1). Data were acquired using a 7300 Real-Time PCR System with a 7300 System SDS Software (Applied Biosystems). The relative gene expression was calculated with the ΔΔCt method using 18*S* ribosomal RNA for normalization (*62*). Statistical analysis was conducted on linear ΔCt values. Data are visualized using GraphPad Prism version 11.0.

### Ruxolitinib treatment

BEpCs from donor 2 were pretreated basally for 86 hours before infection with either 5 μM ruxolitinib (Santa Cruz Biotechnology, no. sc-364729) or 5 μM dimethyl sulfoxide (DMSO; Sigma-Aldrich, no. 41640-100ML) as a control.

### Viral growth curve

BEpCs were infected with respective viruses and MOIs as described above. At indicated time points postinfection, 60 μl of PBSi was added apically to the cells for 5 min at 37°C and then collected and stored at −80°C. Virus titers were determined by plaque assay on MDCK cells. Data are visualized using GraphPad Prism version 11.0.

### Glycan array of H1N1 viruses

Viral stocks were inactivated using 0.03% PFA (Electron Microscopy Sciences, no. 15710) for 4 days at 4°C. For HA titer determination, viral stocks were serially diluted twofold in PBS and incubated with 0.5% chicken red blood cells. An HA titer of 1:128 to 1:512 was used. Consequently, the viral isolates were diluted in PBS-T (PBS and 0.1% Tween 20) and applied to subarrays in the presence of oseltamivir (200 nM) in a humidified chamber for 1 hour. Next, the microarray slide was rinsed with PBS-T, PBS, and deionized water (twice) and dried by centrifugation. The slide was incubated for 1 hour in the presence of the CR6261 stem-specific antibody (100 μl, 5 μg/ml^−1^ in PBS-T) (*32*) and washed as described above. A secondary goat antihuman Alexa Fluor-647 antibody (100 μl, 2 μg/ml^−1^ in PBS-T) (Thermo Fisher Scientific, no. A-21445) was applied, and the resulting slide was incubated for 1 hour in a humidified chamber and then washed by the standard procedure. Afterward, the slides were rinsed successively with PBS-T, PBS, and deionized water. Slides were dried by centrifugation after the washing steps and scanned immediately using an Innopsys Innoscan 710 microarray scanner. Various gains and PMT values were used to ensure the signals were in the linear range and to avoid saturation of the signals. Images were analyzed with Mapix software (version 8.1.0 Innopsys) and processed with an Excel macro (https://github.com/enthalpyliu/carbohydrate-microarray-processing). The average fluorescence intensity and SD were determined for each compound after removal of the highest and lowest intensities from the spot replicates to give *n* = 4. Data are visualized using GraphPad Prism version 11.0.
