# Supplementary material for: Opposing cell-type preferences for binding and replication shape influenza A virus infection in human airways
Source: Sci Adv. 2026 Oct 23;12(43):eaei5782. doi: 10.1126/sciadv.aei5782 (PMC13693689; doi:10.1126/sciadv.aei5782)
Supplement: Supplementary file 1 [file sciadv.aei5782_sm.pdf]

Science Advances
AAAS

Supplementary Materials for

Opposing cell-type preferences for binding and replication shape influenza A virus infection in human airways

Josephine von Kempis *et al.*

Corresponding author: Silke Stertz, stertz.silke@virology.uzh.ch

Sci. Adv. **12**, eaei5782 (2026)
DOI: 10.1126/sciadv.aei5782

This PDF file includes:

Figs. S1 to S11
Table S1

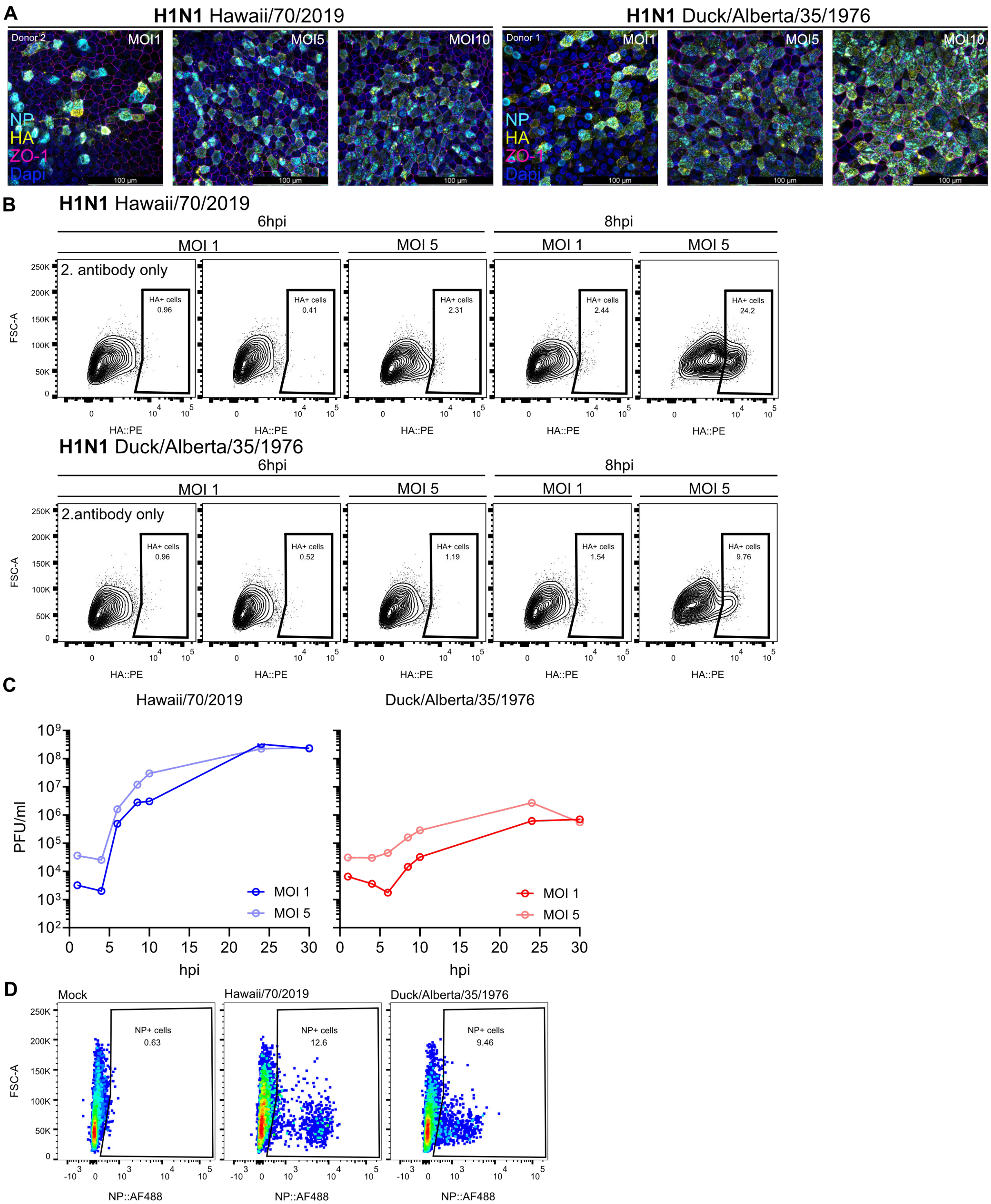

Fig. S1. Flow cytometry and microscopy analysis reveal divergent bronchial epithelial cell tropism of human and avian influenza A viruses. A) Representative microscopy images of the samples described in Fig. 1C. BEpC from donors 1 or 2 were infected with H1N1 A/Duck/Alberta/35/1976 or A/Hawaii/70/2019, respectively, with a multiplicity of infection (MOI) of 1, 5, or 10. At 8 hours post-infection (hpi), the cells were fixed and stained for viral nucleoprotein (anti-NP antibody), viral hemagglutinin (anti-HA antibody), tight junctions (anti-ZO-1 antibody), and nuclei (DAPI). **B)** BEpC from donor 3 were infected with H1N1

Duck/Alberta/35/1976 or Hawaii/70/2019 at an MOI of 1 or 5 for 6 to 8 hours. Cells were prepared for flow cytometry. Cellular doublets were excluded by size, and dead cells using a cell viability marker. The percentage of infected cells was measured by gating for cells with surface hemagglutinin (HA) expression. Secondary antibody only staining was used as a negative control. **C)** BEpC from donor 3 were infected with H1N1 A/Duck/Alberta/35/1976 or A/Hawaii/70/2019, with an MOI of 1 or 5. At the indicated time points post-infection, the apical viral release was harvested and plaqued in MDCK cells. **D)** Representative dot plots of the gating strategy of NP-positive cells for Fig. 1G. BEpC from donor 2 were infected with Hawaii/70/2019 or Duck/Alberta/35/1976 (MOI 5). At 7 hpi, cells were prepared for flow cytometry and stained for infected cells (NP-positive cells). In B) - C), data represent n=1 experiment per infection.

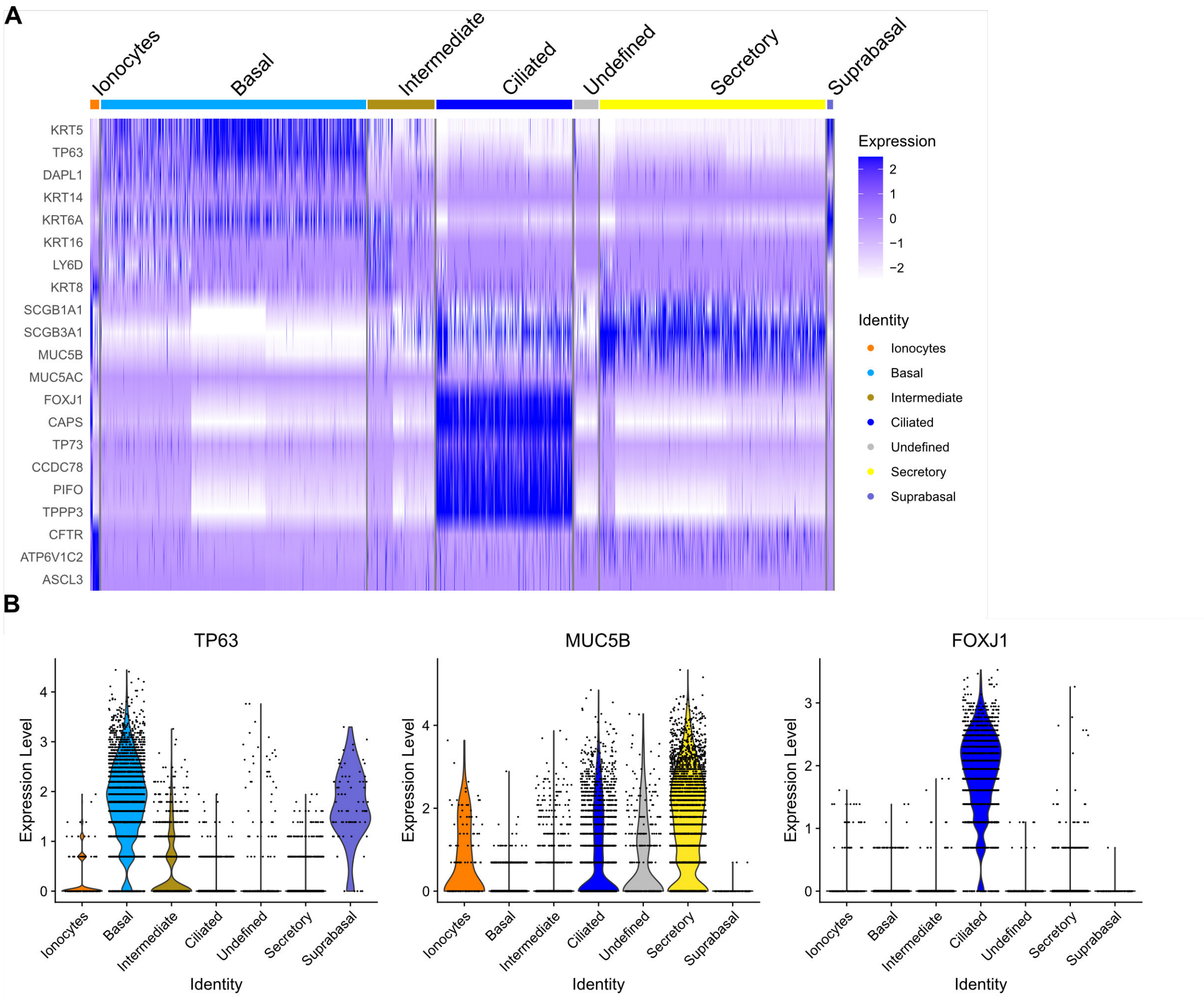

Fig. S2. Defining single-cell sequencing cell cluster identity via cell cluster markers. A) Heatmap depicting single-cell transform (SCTransform) -normalized expression of a set of canonical subtype-specific markers in the single-cell RNA sequencing (scRNAseq) data set from bronchial donors 1-3 mock-infected, described in Fig. 2A. Cell subtype allocation was based on the expression of canonical subtype-specific markers, with a minimum of three markers (two for suprabasal cells) required for cluster allocation. Basal cells (light blue): TP63, KRT5, DAPL1, KRT15, ITGA6, KRT17; Secretory cells (yellow): SCGB3A1, SCGB1A1, MUC5B, MUC5AC, SPDEF, TCN1, BPIFB1, SPRR3, AGR2; Ciliated cells (dark blue): FOXJ1, CAPS, TP73, CCDC78; Ionocytes (orange): CFTR, ASCL3, FOXI1, ATP6V1C2. Suprabasal cells (violet): KRT6A and KRT15. Intermediate cells (brown): clusters expressing some markers of basal and secretory cells. Undefined cells (grey): clusters not assignable to known epithelial subtype. **B)** Violin plots depicting SCTransform-normalized expression of TP63, MUC5B, and FOXJ1 within each subtype of the scRNAseq data set from bronchial mock-infected cells (donor 1-3 pooled). In A) - B), data represent n=1 experiment per donor (donor 1-3).

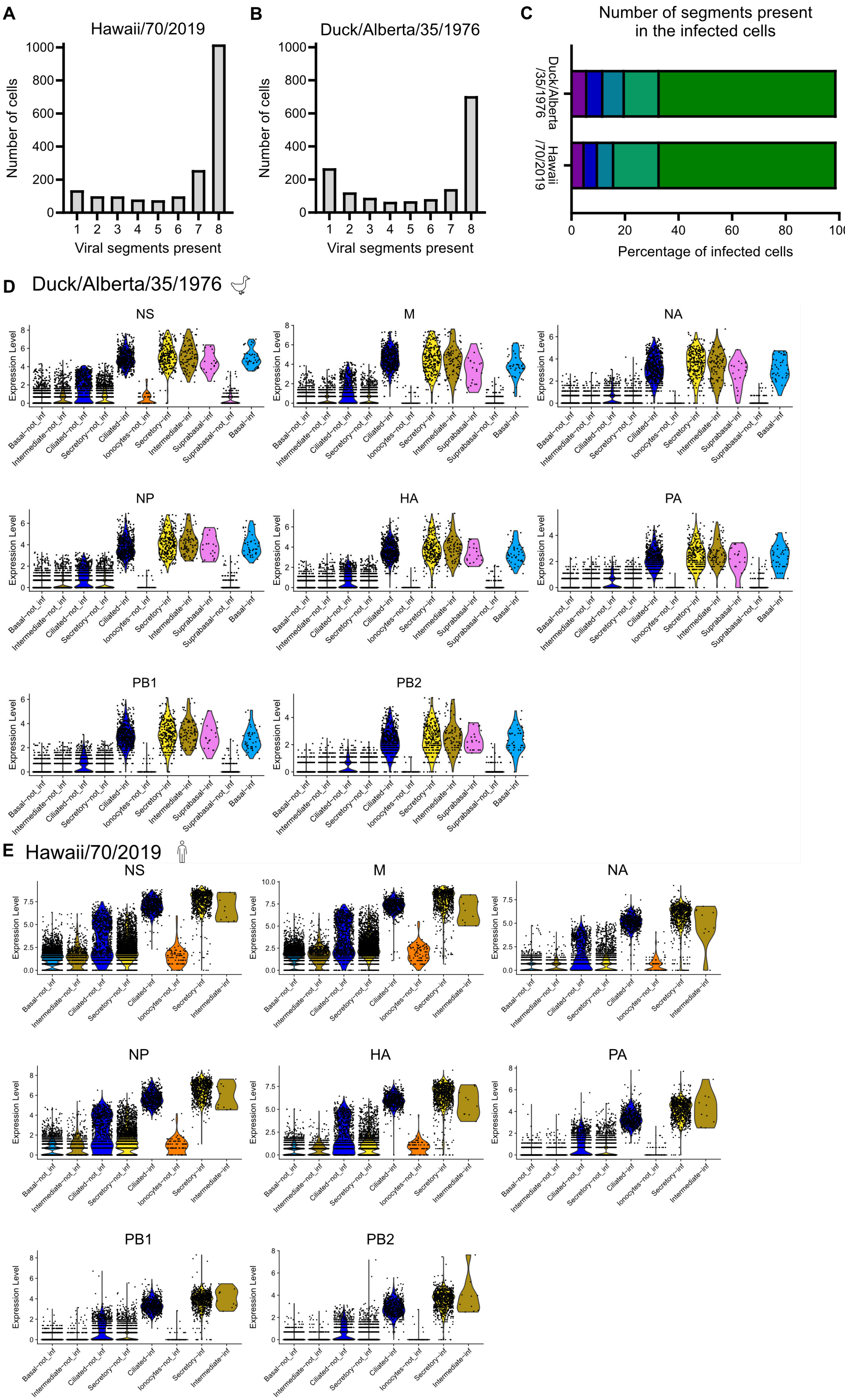
A
Hawaii/70/2019
Number of cells
Viral segments present
B
Duck/Alberta/35/1976
Number of cells
Viral segments present
C
Number of segments present in the infected cells
Duck/Alberta /35/1976
Hawaii /70/2019
Percentage of infected cells
8
7
6
5
4
D
Duck/Alberta/35/1976
NS
M
NA
NP
HA
PA
PB1
PB2
Expression Level
Basal−not_inf
Intermediate−not_inf
Ciliated−not_inf
Secretory−not_inf
Ciliated−inf
Ionocytes−not_inf
Secretory−inf
Intermediate−inf
Suprabasal−inf
Suprabasal−not_inf
Basal−inf
E
Hawaii/70/2019
NS
M
NA
NP
HA
PA
PB1
PB2
Expression Level
Basal−not_inf
Intermediate−not_inf
Ciliated−not_inf
Secretory−not_inf
Ciliated−inf
Ionocytes−not_inf
Secretory−inf
Intermediate−inf

Fig. S3. Viral segments expression in single-cell sequencing dataset. A) Number of cells expressing 1 to 8 Hawaii/70/2019 viral segments in scRNAseq dataset. **B)** Number of cells expressing 1 to 8 Duck/Alberta/35/1976 viral segments in scRNAseq dataset. **C)** Cellular composition of influenza A virus infected cells expressing 4 to 8 viral segments. **D)** Violin plots depicting SCTransform-normalized expression of Duck/Alberta/35/1976 viral segments within each cell subtype of the scRNAseq dataset (donor 1-3 pooled). **E)** Violin plots depicting SCTransform-normalized expression of Hawaii/70/2019 viral segments within each cell subtype of the scRNAseq dataset (donor 1-3 pooled). The Hawaii/0/2019 infected samples did not present suprabasal cells, nor infected basal cells, and therefore shows overall less cell subtypes than the Duck/Alberta/35/1976 infected samples presented in D). In A) - E), data represent n=1 experiment per donor (donor 1-3).

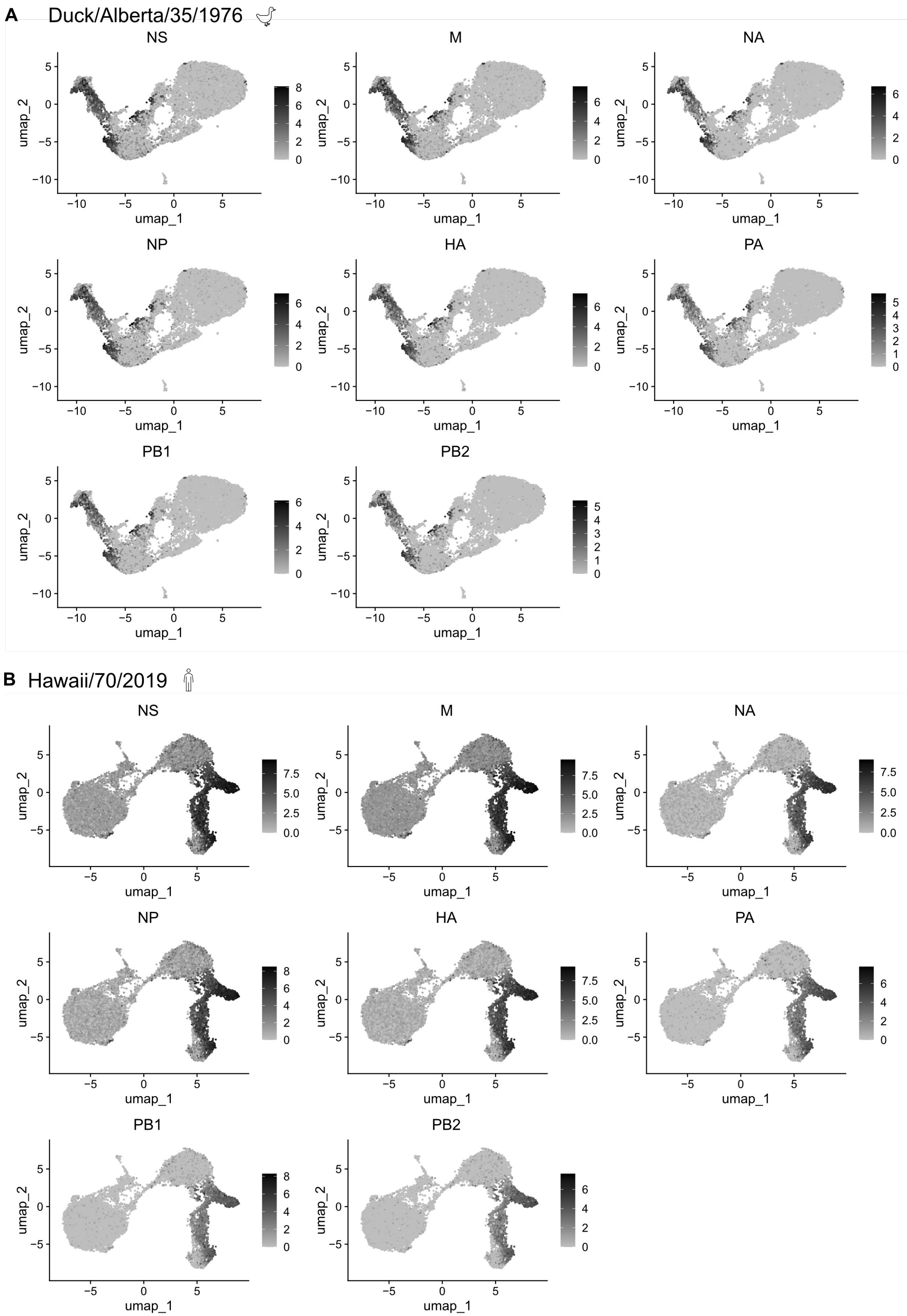

A
Duck/Alberta/35/1976
NS
M
NA
NP
HA
PA
PB1
PB2
umap_1
umap_2
B
Hawaii/70/2019
NS
M
NA
NP
HA
PA
PB1
PB2
umap_1
umap_2

Fig. S4. Viral segments expression in single-cell sequencing dataset. A) UMAP visualization of SCTransform-normalized expression of Duck/Alberta/35/1976 viral segments (donor 1-3 pooled). **B)** UMAP visualization of SCTransform-normalized expression of Hawaii/70/2019 viral segments (donor 1-3 pooled). In A) - B), data represent n=1 experiment per donor (donor 1-3).

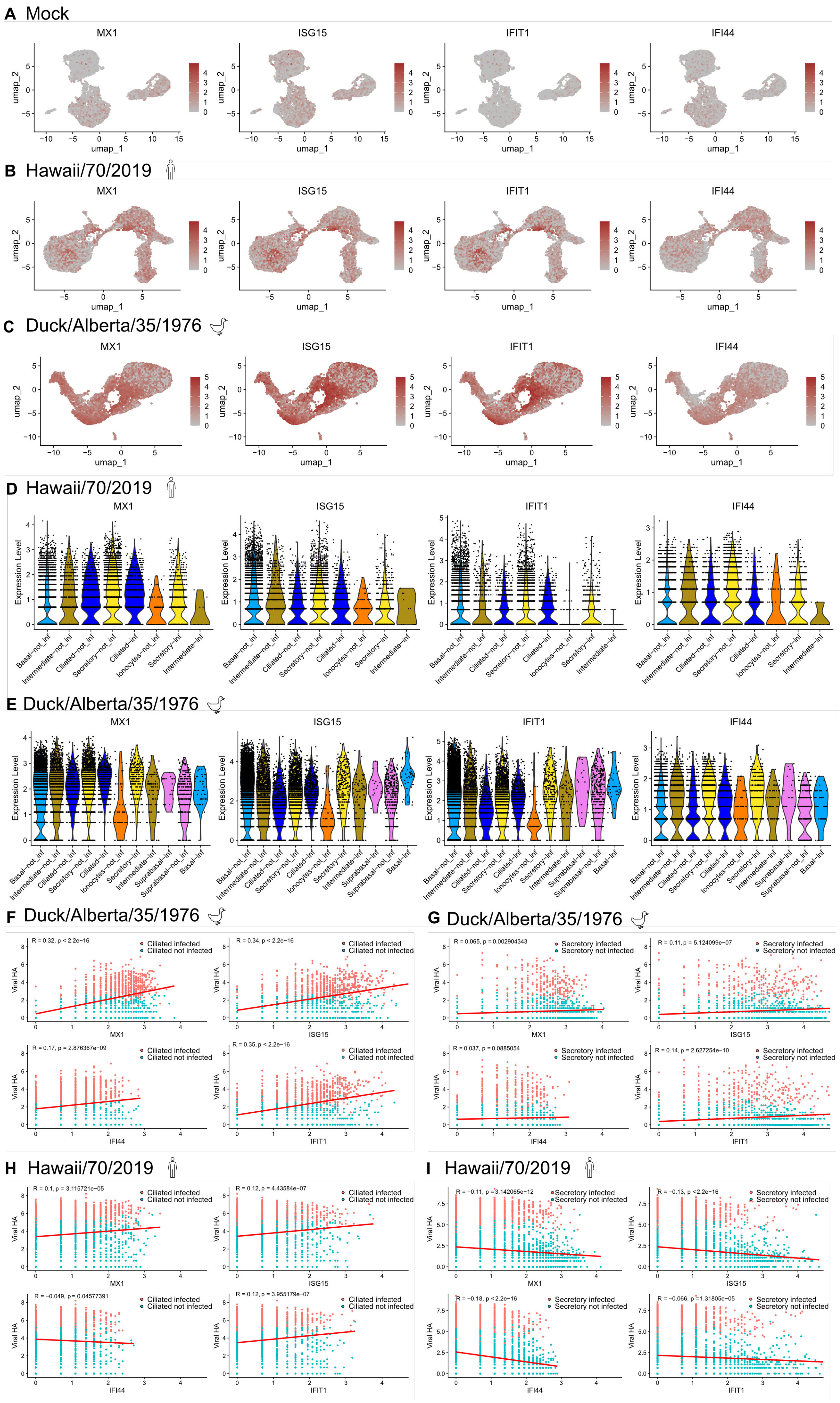
A Mock
MX1
ISG15
IFIT1
IFI44
umap_1
umap_2
B Hawaii/70/2019
C Duck/Alberta/35/1976
D Hawaii/70/2019
Expression Level
Basal–not_inf
Intermediate–not_inf
Ciliated–not_inf
Secretory–not_inf
Ciliated–inf
Ionocytes–not_inf
Secretory–inf
Intermediate–inf
E Duck/Alberta/35/1976
Suprabasal–inf
Suprabasal–not_inf
Basal–inf
F Duck/Alberta/35/1976
R = 0.32, p < 2.2e−16
R = 0.34, p < 2.2e−16
R = 0.17, p = 2.876367e−09
R = 0.35, p < 2.2e−16
Ciliated infected
Ciliated not infected
Viral HA
G Duck/Alberta/35/1976
R = 0.065, p = 0.002904343
R = 0.11, p = 5.124099e−07
R = 0.037, p = 0.0885054
R = 0.14, p = 2.627254e−10
Secretory infected
Secretory not infected
H Hawaii/70/2019
R = 0.1, p = 3.115721e−05
R = 0.12, p = 4.43584e−07
R = −0.049, p = 0.04577391
R = 0.12, p = 3.955179e−07
I Hawaii/70/2019
R = −0.11, p = 3.142065e−12
R = −0.13, p < 2.2e−16
R = −0.18, p < 2.2e−16
R = −0.066, p = 1.31805e−05

Fig. S5. Early influenza A virus transcription and replication efficiency is independent of interferon-stimulated gene expression. A) - C) UMAP visualization of SCTransform-normalized expression of MX1, ISG15, IFIT1, and IFI44 in A) mock-infected, B) Hawaii/70/2019 infected, C) Duck/Alberta/35/1976 infected samples (donor 1-3 pooled). **D) - E)** Violin plots depicting SCTransform-normalized expression of MX1, ISG15, IFIT1, and IFI44 in D) Hawaii/70/2019 infected and E) Duck/Alberta/35/1976 infected samples (donor 1-3 pooled). The Hawaii/0/2019 infected samples shown in D) did not present suprabasal cells, nor infected basal cells, and therefore shows overall less cell subtypes than the Duck/Alberta/35/1976 infected samples presented in E). **F) - I)** Correlation plot of SCTransform-normalized expression of MX1, ISG15, IFIT1, and IFI44 in F) - G) Duck/Alberta/35/1976 and H) - I) Hawaii/70/2019 infected samples (donor 1-3 pooled). Linear regression line added in red and correlation coefficient (R) and *p*-value were calculated. In A) - I), data represent n=1 experiment per donor (donor 1-3).

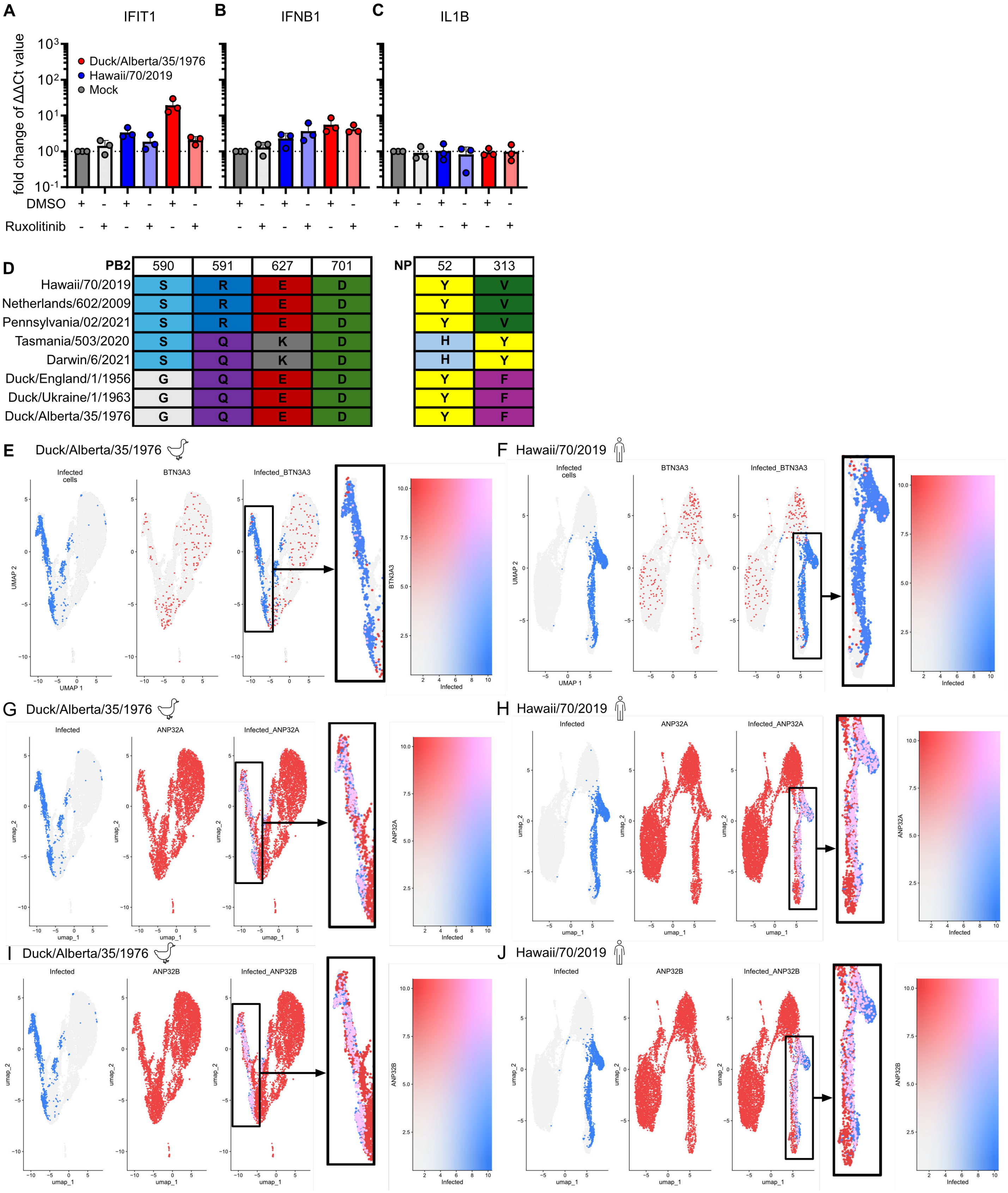
A
IFIT1
B
IFNB1
C
IL1B
fold change of ΔΔCt value
Duck/Alberta/35/1976
Hawaii/70/2019
Mock
DMSO
Ruxolitinib
D
PB2
590
591
627
701
NP
52
313
Hawaii/70/2019
Netherlands/602/2009
Pennsylvania/02/2021
Tasmania/503/2020
Darwin/6/2021
Duck/England/1/1956
Duck/Ukraine/1/1963
Duck/Alberta/35/1976
E
Duck/Alberta/35/1976
F
Hawaii/70/2019
Infected cells
BTN3A3
Infected_BTN3A3
UMAP 1
UMAP 2
Infected
G
Duck/Alberta/35/1976
H
Hawaii/70/2019
ANP32A
Infected_ANP32A
umap_1
umap_2
I
Duck/Alberta/35/1976
J
Hawaii/70/2019
ANP32B
Infected_ANP32B

Fig. S6. Early influenza A virus replication efficiency is dependent on ANP32 compatibility. **A) - C)** BEpC from donor 2 were pretreated with DMSO (control) or ruxolitinib for 86 hours prior to infection. The cells were infected with mock, Hawaii/70/2019, or Duck/Alberta/35/1976 (MOI 1). At 6 hpi, the RNA was extracted and analysed by RT-qPCR. The ΔCt was calculated using 18s-rRNA for normalization. The enrichment (ΔΔCt) of the transcript levels of A) IFIT1, B) IFNB, and C) IL1B are presented as a fold change relative to mock DMSO control. **D)** Amino acid sequence alignment of PB2 and NP highlighted positions (PB2 590, 591, 627, 701, and NP 52, 313) across viruses (Hawaii/70/2019, Netherlands/602/2009, Pennsylvania/02/2021, Tasmania/503/2020, Darwin/6/2021, Duck/England/1/1956, Duck/Ukraine/1/1963, and Duck/Alberta/35/1976). Amino acid numbering based on alignment with Hawaii/70/2019 sequence. **E)** UMAP visualization of Duck/Alberta/35/1976 infected cells and BTN3A3 expression (donor 1-3 pooled). **F)** UMAP visualization of Hawaii/70/2019 infected cells and BTN3A3 expression (donor 1-3 pooled). **G)** UMAP visualization of Duck/Alberta/35/1976 infected cells and ANP32A expression (donor 1-3 pooled). **H)** UMAP visualization of Hawaii/70/2019 infected cells and ANP32A expression (donor 1-3 pooled). **I)** UMAP visualization of Duck/Alberta/35/1976 infected cells and ANP32B expression (donor 1-3 pooled). **J)** UMAP visualization of Hawaii/70/2019 infected cells and ANP32B expression (donor 1-3 pooled). In A) - C), data represent means ± s.d. from n=3 independent experiments. In E) - J), data represent n=1 experiment per donor (donor 1-3).

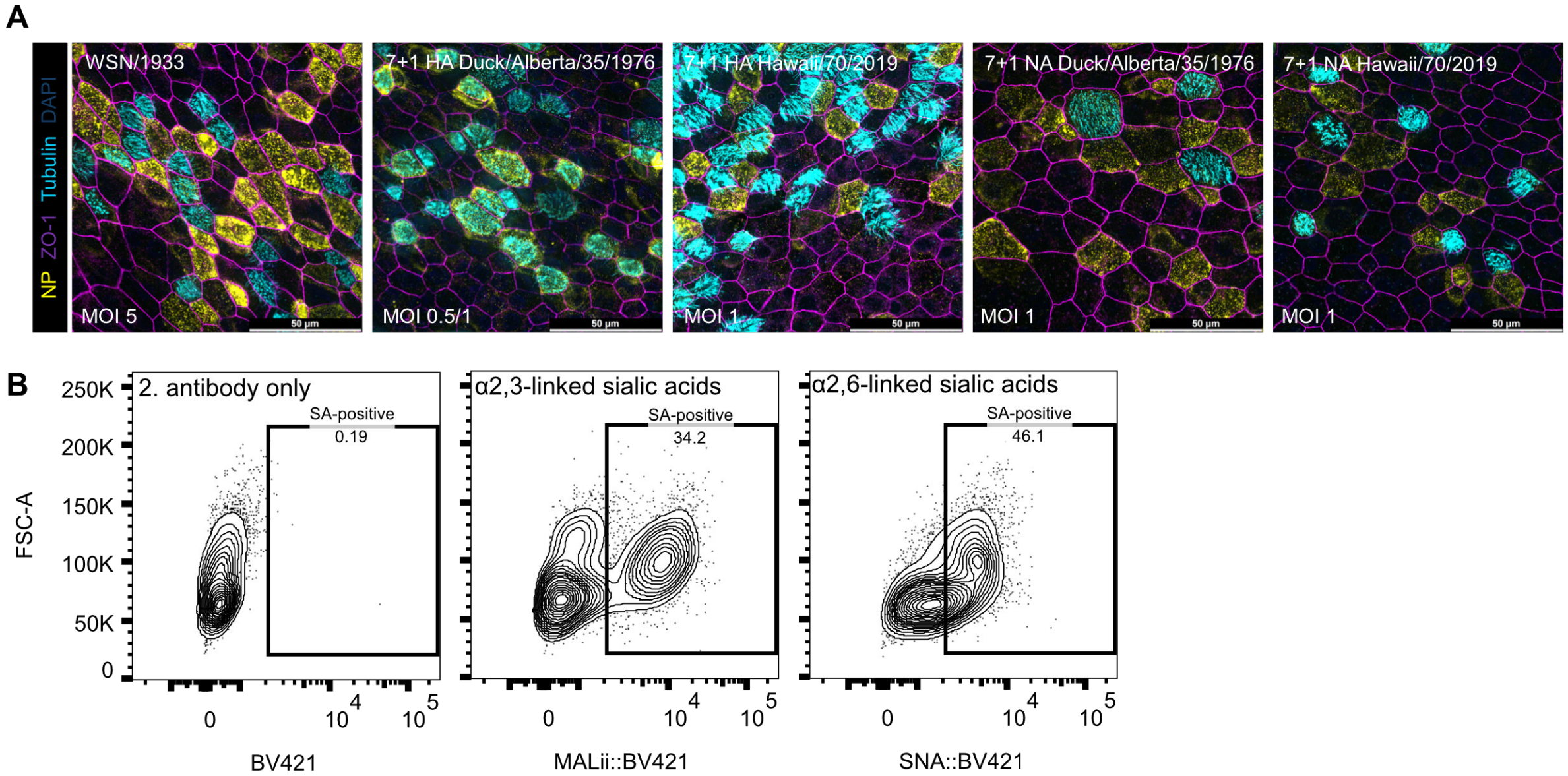

Fig. S7. Influenza A virus tropism in human bronchial epithelial cells is mediated by haemagglutinin and the apical distribution of sialic acid linkages. A) Representative microscopy images of the samples described in Fig. 4A. BEpC from donor 2 were infected with control H1N1 WSN/1933 virus or recombinant 7+1 viruses containing 7 genes from the WSN/1933 strain and either the HA- or NA-encoding segment of Hawaii/70/2019, or of Duck/Alberta/35/1976. An MOI of 0.5-1 was used for the recombinant strains, and an MOI of 5 was used for the control WSN/1933 strain to achieve comparable infection rates. 7 hpi, the cells were fixed and stained for viral nucleoproteins (anti-NP antibody), ciliated cells (microtubule dye), tight junctions (anti-ZO-1 antibody), and nuclei (DAPI). **B)** Representative dot plots of gating strategy to determine sialic acid positive cells for Fig. 4, B to G. BEpC from donor 2 were trypsinized and stained for cell surface markers identifying ciliated cells (microtubules), basal cells (CD271), secretory cells (CD66c), α2,3-linked sialic acids (Maackia amurensis II lectins; MALii), and α2,6-linked sialic acids (Sambucus nigra lectins; SNA). The cells were analyzed by flow cytometry, cellular doublets were excluded by size, and dead cells with a cell viability marker.

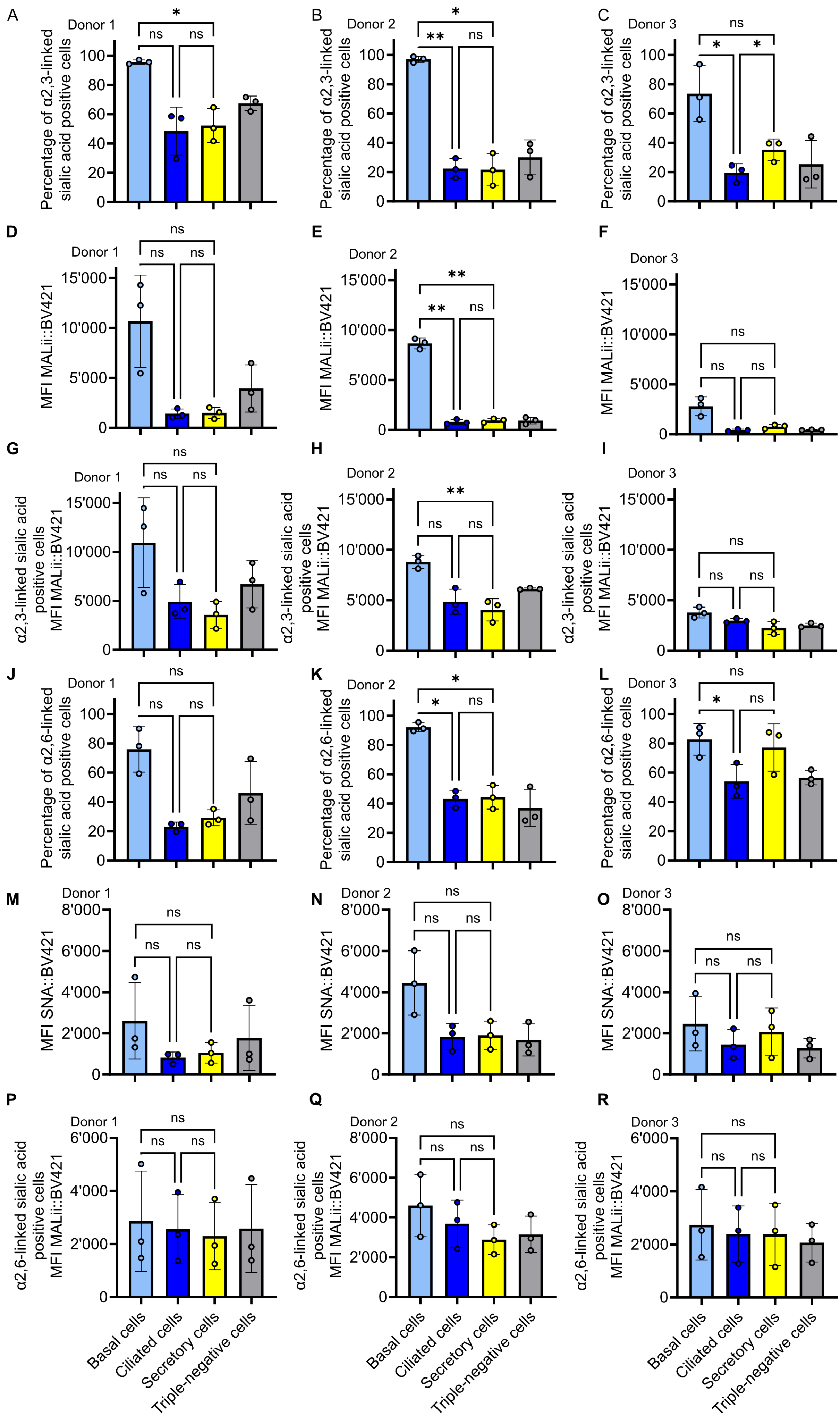

A
B
C
D
E
F
G
H
I
J
K
L
M
N
O
P
Q
R
Donor 1
Donor 2
Donor 3
Percentage of α2,3-linked sialic acid positive cells
MFI MALii::BV421
α2,3-linked sialic acid positive cells MFI MALii::BV421
Percentage of α2,6-linked sialic acid positive cells
MFI SNA::BV421
α2,6-linked sialic acid positive cells MFI MALii::BV421
ns
*
**
Basal cells
Ciliated cells
Secretory cells
Triple-negative cells

Fig. S8. Influenza A virus tropism in human bronchial epithelial cells is mediated by haemagglutinin and the apical distribution of sialic acid linkages. A) - R) BEpC from donor 1-3 were trypsinized and stained for cell surface markers identifying ciliated cells (microtubules), basal cells (CD271), secretory cells (CD66c), α2,3-linked sialic acids (Maackia amurensis II lectins; MALii), and α2,6-linked sialic acids (Sambucus nigra lectins; SNA). The cells were analyzed by flow cytometry, cellular doublets were excluded by size, and dead cells were excluded with a cell viability marker. A) - C) and J) - L) show the percentage of α2,3-linked and α2,6-linked sialic acid-positive cells, respectively. D) - F) and M) - O) show the median fluorescent intensity (MFI) of the BV421 staining of α2,3-linked and α2,6-linked sialic acids, respectively. G) - I) and P) - R) show the MFI of the BV421 staining of α2,3-linked and α2,6-linked sialic acids, respectively, of pre-gated sialic acid-positive cells. One repeat was previously published in (*45*). In A) - R), data represent means ± s.d. from n = 3 independent experiments. Statistical significance was determined in A) - R) using RM one-way ANOVA with Tukey's multiple comparisons test. $^{*}P \leq 0.05$, $^{**}P \leq 0.01$.

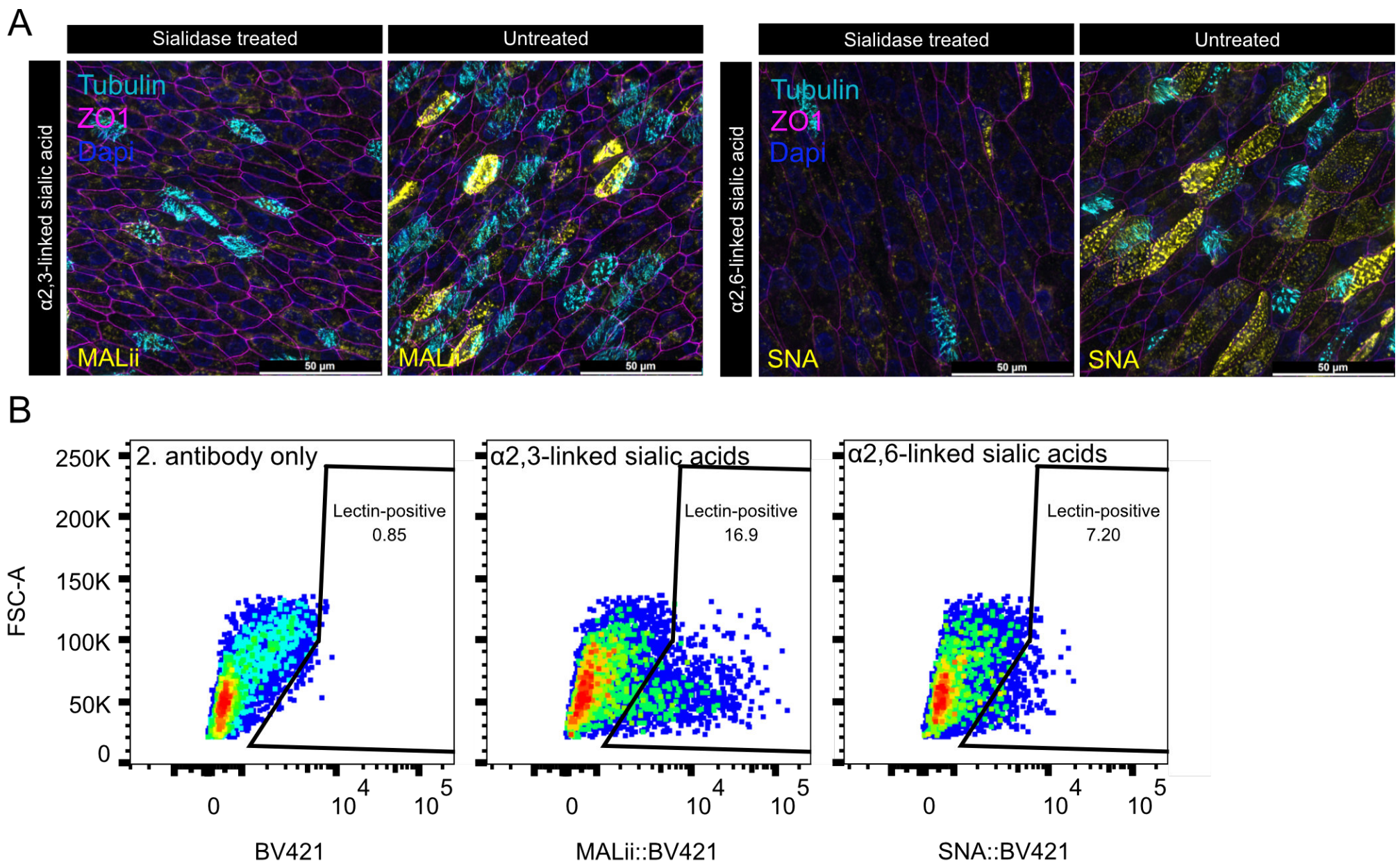

Fig. S9. Influenza A virus tropism in human bronchial epithelial cells is mediated by haemagglutinin and the apical distribution of sialic acid linkages. A) Representative microscopy images of apical sialic acid stainings on BEpC from donor 2 (untreated or sialidase-treated). The cells were fixed and stained for surface α2,3-linked sialic acids (Maackia amurensis II lectins; MALii) or α2,6-linked sialic acid (Sambucus nigra lectins; SNA), as well as for tight junctions (anti-ZO-1 antibody), ciliated cells (anti-beta IV tubulin antibody), and nuclei (DAPI). **B)** Representative dot plots of gating strategy to determine apically sialic acid positive cells for Fig. 4, H to I. BEpC from donor 2 were stained prior to trypsinization for apical α2,3-linked sialic acids (MALii) and α2,6-linked sialic acid (SNA), as well as ciliated cells (microtubule dye). The cells were analyzed by flow cytometry, cellular doublets were excluded by size, and dead cells with a cell viability marker.

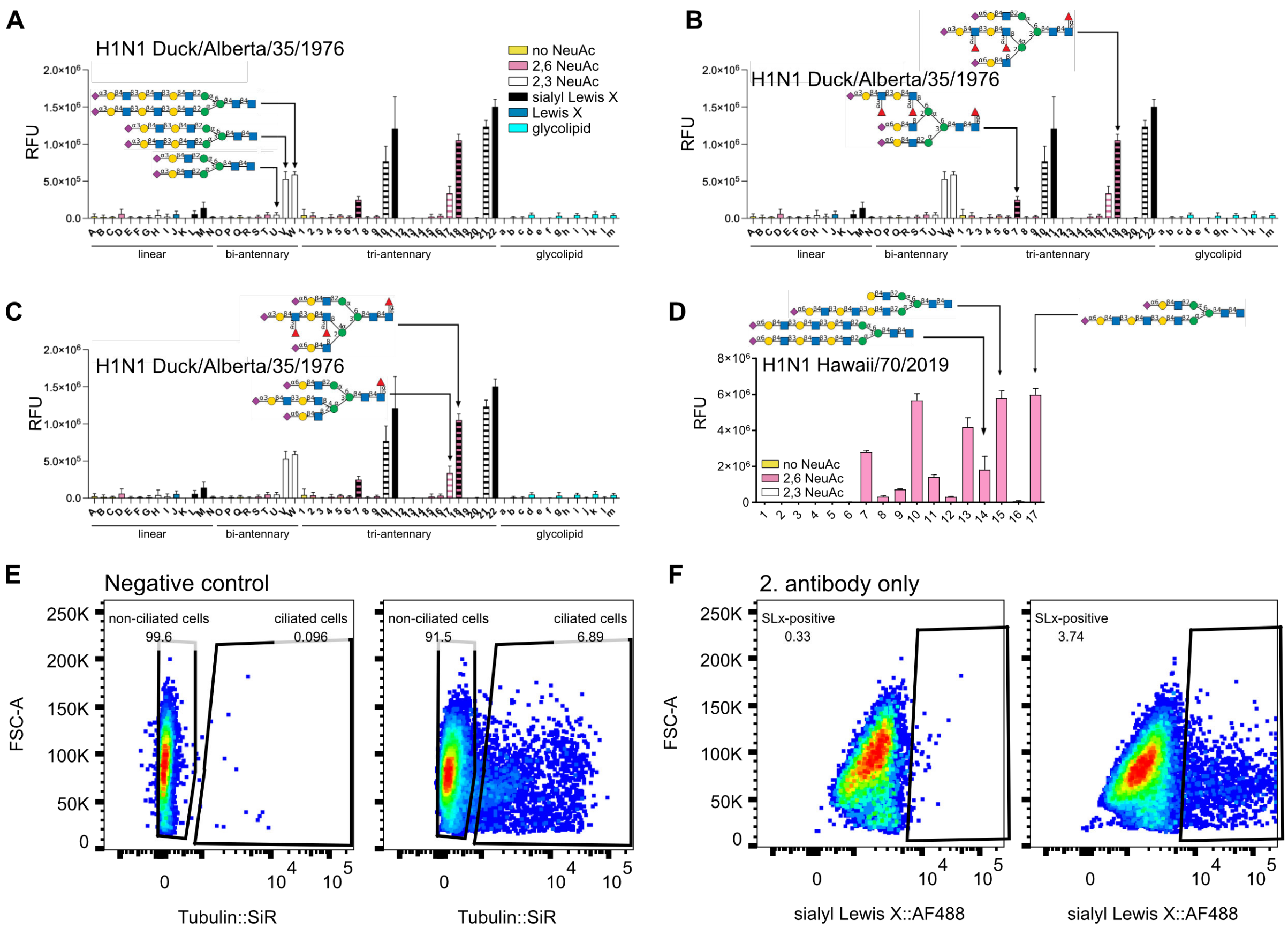

Fig. S10. Avian H1N1 preference for sialyl Lewis X correlates with glycan abundance on ciliated cells. A) - C) Microarray binding of H1N1 Duck/Alberta/35/1976 with glycans of Fig. 5A. The microarray tested for glycans with no sialylation (yellow), α2,6-linked NeuAc (pink), α2,3-linked NeuAc (white), Lewis X (blue), sialyl Lewis X (black), and glycolipids (cyan). Striped bars indicate glycans terminating in different epitopes on different arms. Arrows highlight specific results. **D)** Microarray binding of H1N1 Hawaii/70/2019 with glycans of Fig. 5F. The microarray tested for glycans with no sialylation (yellow), α2,6-linked NeuAc (pink), and α2,3-linked NeuAc (white). **E) - F)** Representative dot plots of gating strategy to determine E) non-/ciliated cells and F) apically sialyl Lewis X positive cells for Fig. 5D. BEpC from donor 2 were stained before trypsinization for apical sialyl Lewis X and ciliated cells (microtubule dye). The cells were analyzed by flow cytometry, cellular doublets were excluded by size, and dead cells with a cell viability marker.

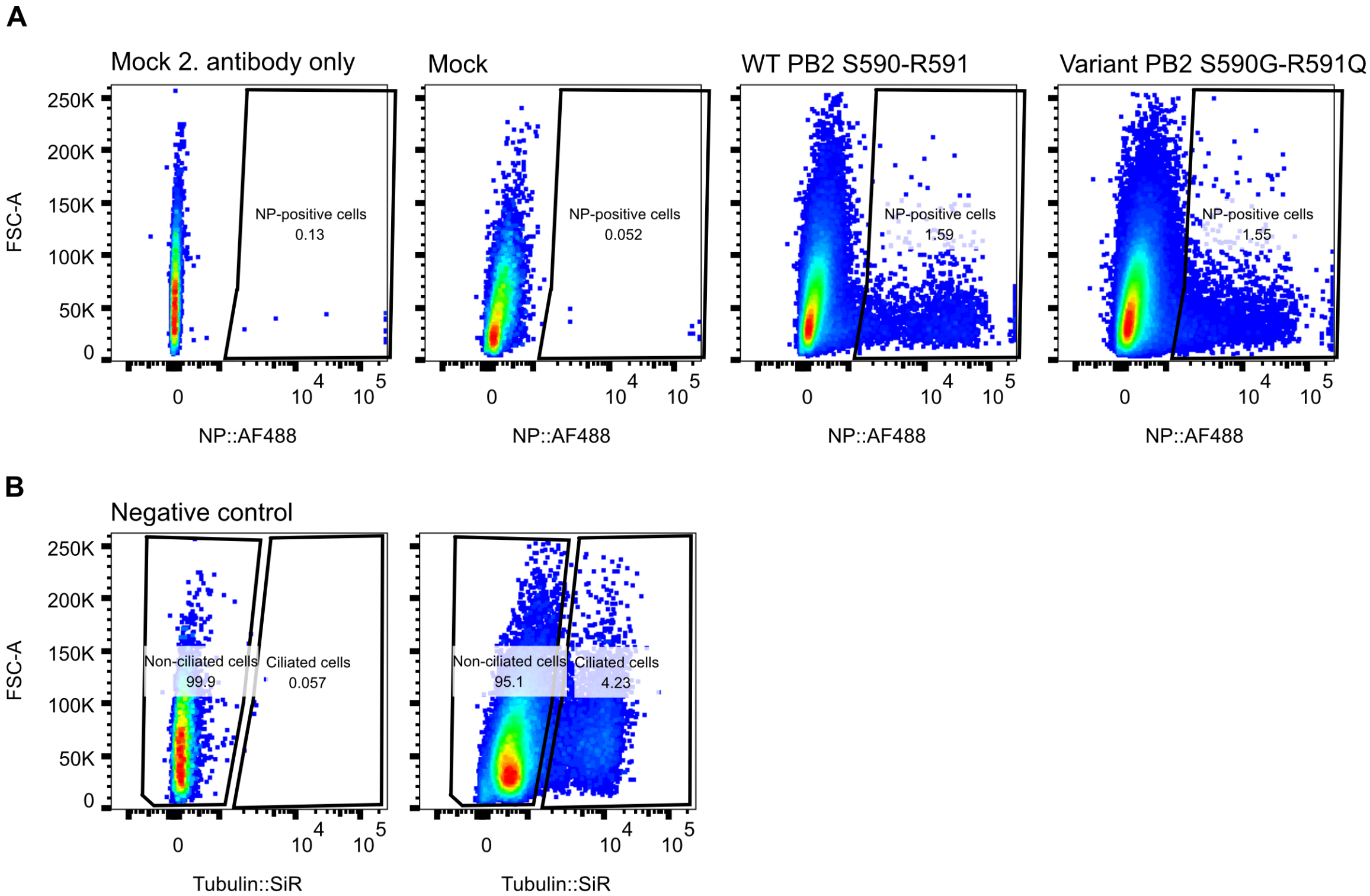

Fig. S11. Early viral RNA and protein levels of human H1N1 are cell subtype specific. A) - B) Representative dot plots of the gating strategy of A) NP-positive cells and B) ciliated cells for Fig. 6I. BEpC from donor 2 were infected with Netherlands/602/2009 PB2 S590-R591 or with Netherlands/602/2009 PB2 S590G-R591Q (MOI of 0.45). At 8 hpi, cells were prepared for flow cytometry and stained for infected cells (NP-positive cells) and ciliated cells (microtubule dye). The cells were analyzed by flow cytometry, cellular doublets were excluded by size, and dead cells were excluded with a cell viability marker.

IAV M (WHO M30F2) primer	ATGAGYCTTYTAACCGAGGTCGAAACG
IAV M (WHO M264R3) primer	TGGACAAANCGTCTACGCTGCAG
18s-rRNA-F primer	GGCCCTGTAATTGGAATGACTC
18s-rRNA-R primer	CCAAGATCCAACTACGAGCTT
Universal flu primer 3'NCAMBIA	GATCGCTCTTCTGGGAGCAAAAGCAGG
Universal flu primer 5'NCAMBI	CATCGCTCTTCTATTAGTAGAAACAAGG
InFusion-Uni-Flu-1F	CCGAAGTTGGGGGGGAGCGAAAGCAGG
InFusion-Uni-Flu-R	GGCCGCCGGGTTATTAGTAGAAACAAGG
NL09-PB2-GQ-1R	CCACTGTATTGACCTCTGGTTGCCTT
NL09-PB2-GQ-1F	AAGGCAACCAGAGGTCAATACAGTGG
MX1 Forward	AGACAAGGTTGTGGACGTGG
MX1 Reverse	TTCCTCCAGCAGATCCCTGA
IFIT2 Forward	ACTGCAACCATGAGTGAGAAC
IFIT2 Reverse	GATAGGCCAGTAGGTTGCACA
ISG15 Forward	TGTCGGTGTCAGAGCTGAAG
ISG15 Reverse	GCCCTTGTTATxCCTCACCA
IFIT1 Forward	CTGTGGTAGGCTCTGCTTCC
IFIT1 Reverse	CCACCACACCCAGCTAAGTT
IFNB1 Forward	CCTGTGGCAATTGAATGGGA
IFNB1 Reverse	CTCATAGATGGTCAATGCGGC
IL1B Forward	AGCTCGCCAGTGAAATGATG
IL1B Reverse	TCGGAGATTCGTAGCTGGATG

Table S1. Primer sequences.
